# New 2-Ethylthio-4-methylaminoquinazoline derivatives inhibiting two subunits of cytochrome *bc1* in *Mycobacterium tuberculosis*

**DOI:** 10.1371/journal.ppat.1008270

**Published:** 2020-01-23

**Authors:** Andréanne Lupien, Caroline Shi-Yan Foo, Svetlana Savina, Anthony Vocat, Jérémie Piton, Natalia Monakhova, Andrej Benjak, Dirk A. Lamprecht, Adrie J. C. Steyn, Kevin Pethe, Vadim A. Makarov, Stewart T. Cole

**Affiliations:** 1 Global Health Institute, Ecole Polytechnique Fédérale de Lausanne, Lausanne, Switzerland; 2 Department of Stresses of Microorganisms, A. N. Bach Institute of Biochemistry, Moscow, Russian Federation; 3 Africa Health Research Institute, Durban, South Africa; 4 Department of Microbiology, University of Alabama at Birmingham, Birmingham, Alabama, United States of America; 5 Lee Kong Chian School of Medicine and School of Biological Sciences, Nanyang Technological University, Singapore; 6 Institut Pasteur, rue du Docteur Roux, France; University of Massachusetts Medical School, UNITED STATES

## Abstract

The emergence of multi-drug (MDR-TB) and extensively-drug resistant tuberculosis (XDR-TB) is a major threat to the global management of tuberculosis (TB) worldwide. New chemical entities are of need to treat drug-resistant TB. In this study, the mode of action of new, potent quinazoline derivatives was investigated against *Mycobacterium tuberculosis* (*M*. *tb*). Four derivatives 11626141, 11626142, 11626252 and 11726148 showed good activity (MIC ranging from 0.02–0.09 μg/mL) and low toxicity (TD_50_ ≥ 5μg/mL) *in vitro* against *M*. *tb* strain H37Rv and HepG2 cells, respectively. 11626252 was the most selective compound from this series. Quinazoline derivatives were found to target cytochrome *bc*_*1*_ by whole-genome sequencing of mutants selected with 11626142. Two resistant mutants harboured the transversion T943G (Trp312Gly) and the transition G523A (Gly175Ser) in the cytochrome *bc*_*1*_ complex cytochrome *b* subunit (QcrB). Interestingly, a third mutant QuinR-M1 contained a mutation in the Rieske iron-sulphur protein (QcrA) leading to resistance to quinazoline and other QcrB inhibitors, the first report of cross-resistance involving QcrA. Modelling of both QcrA and QcrB revealed that all three resistance mutations are located in the stigmatellin pocket, as previously observed for other QcrB inhibitors such as Q203, AX-35, and lansoprazole sulfide (LPZs). Further analysis of the mode of action *in vitro* revealed that 11626252 exposure leads to ATP depletion, a decrease in the oxygen consumption rate and also overexpression of the cytochrome *bd* oxidase in *M*. *tb*. Our findings suggest that quinazoline-derived compounds are a new and attractive chemical entity for *M*. *tb* drug development targeting two separate subunits of the cytochrome *bc*_*1*_ complex.

## Author summary

Tuberculosis (TB) is the leading cause of death worldwide due to an infectious agent. Nowadays, the efficacy of mainstay anti-TB drugs is jeopardized due to the emergence of drug-resistant TB. New antitubercular drugs are needed for the treatment of TB. In this study, we decipher the mechanism of action of a new potent series of 2-Ethylthio-4-methylaminoquinazoline derivatives against *Mycobacterium tuberculosis* (*M*. *tb*). Out of 76 derivatives tested, 4 compounds (11626141, 11626142, 11626252 and 11726148) have good activity (MIC below 0.09 μg/mL) against *M*. *tb* and low toxicity in human hepatocytes. The lead compound 11626252 and two derivatives, 11626141 and 11626142, were specifically active against members of the *M*. *tb* complex and *M*. *marinum*. These derivatives target the cytochrome *bc*_*1*_ oxidase, part of the mycobacterial electron-transport chain. Interestingly, we demonstrate that resistance to the quinazoline derivatives, as well as to other QcrB inhibitors, like Q203, AX-35 and lansoprazole sulfide, may emerge through a mutation in the Rieske iron-sulphur protein (QcrA). To our knowledge, this is the first report implicating the QcrA subunit in the pharmacological inhibition of cytochrome *bc*_*1*_ activity.

## Introduction

With more than 1.7 million deaths worldwide, including 0.4 million HIV-positive patients, tuberculosis (TB) is the leading cause of death due to a single infectious agent. [1] In 2016, an estimated 10.4 million people fell ill with TB. [1] Treatment of drug-susceptible TB (DS-TB) relies on a combination therapy of isoniazid (INH), rifampicin (RIF), pyrazinamide (PZA) and ethambutol (EMB) for 6 months. Despite the high efficacy of the DS-TB treatment, 490,000 new cases of TB were reported in 2016 to be resistant to both RIF and INH and therefore classified as multidrug-resistant (MDR-TB). [1] In 2016, 6.2% of MDR-TB cases were defined as extensively-resistant TB (XDR-TB) on the basis of their resistance to the main second-line drugs. The estimated treatment success rate for XDR-TB is less than 30%. [1] In light of the current global TB situation, there is an urgent need to improve existing TB treatments through more strategic implementation of existing drugs and/or the introduction of new chemical entities.

Heterocyclic compounds are the backbone of modern medicinal chemistry. This versatile chemical class provides the ability to expand the available drug-like chemical space and drive more efficient delivery of drug discovery programs. [2] The most frequently encountered heterocycles are reported to have strong lipophilic characteristics, which facilitate the permeation of cell membranes. [3] One of the most critical heterocycle families are the benzodiazines, polycyclic compounds containing one or more benzene rings fused to a diazine ring. Derivatives of the quinazoline moiety, also known as 1,3-benzodiazine, were previously shown to have antibacterial, antifungal, anticonvulsant, anti-inflammatory, anti-HIV, anticancer and analgesic activities, with slight modifications of the quinazoline nucleus improving activity. [4] For *M*. *tb*, antimycobacterial activity against strain H37Rv *in vitro* was previously reported for quinazoline 2-carboxylate derivatives, more precisely, thiazoloquinazoline carboxylates. [5] Furthermore, diaminoquinazolines (DAQ) were shown to selectively inhibit *M*. *tb* growth in a range of 1.3–6.1 μg/mL. [6] Although it was discovered that resistant mutants to DAQ harboured a mutation in *rv3161c*, a potential dioxygenase, and that DAQ act as a pro-drug in mycobacteria, the mode of action of these derivatives remains unknown in *M*. *tb*.

We herein describe the structure-activity relationship (SAR) studies of a new series of quinazoline derivatives with improved activity against *M*. *tb in vitro*, *ex vivo* and *in vivo* in an acute model of TB in mice. Quinazoline compounds were found to target cytochrome *bc*_*1*_ in *M*. *tb* and the mechanism of action was confirmed by both genotypic and phenotypic means.

## Results

### Synthesis of quinazoline derivatives

Seventy-six original quinazoline derivatives were synthesized. (S1 Table) Different substitutions were introduced in the phenyl moiety of quinazoline scaffold as well as different amino substitutions in position 4 and different thioalkyls in position 2 of the molecule to study the SAR and to find the most active compounds.

### SAR on the susceptibility of *M*.*tb* to the quinazoline derivatives *in vitro* and *ex vivo*

**Fig 1:**
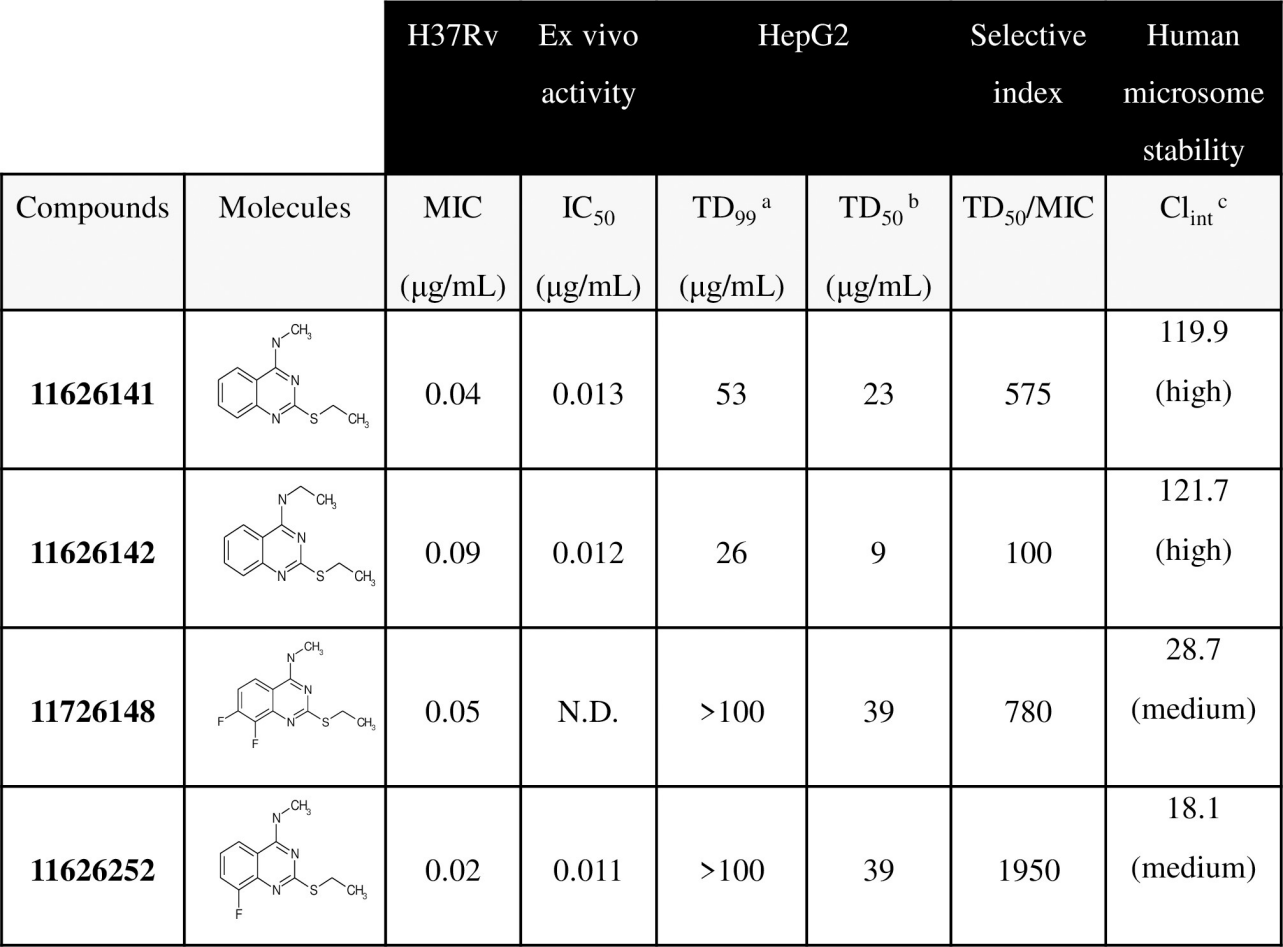
Susceptibility of *M*. and cytotoxicity of HepG2 cells to quinazoline derivatives. ^a^ Toxic dose that inhibits 99% of cell growth.^b^ Toxic dose that inhibits 50% of cell growth.^c^ Intrinsic Clearance (Clint) is expressed in μL/min/mg protein. Abbreviation: N.D. not determined.

Susceptibility to the quinazoline derivatives was first assessed by the resazurin microtiter assay (REMA). (Fig 1) Compounds 11626141 and 11626142 were identified with activity against *M*. *tb* H37Rv *in vitro* during whole-cell screening, and have MICs of 0.05 μg/mL and 0.09 μg/mL, respectively. (Fig 1) As the next step of the synthesis, we systematically changed each proton in the phenyl moiety of the quinazoline scaffold to identify possible sites for future derivatization. Fluorine atoms were found to be preferable in positions 6 and 8 since such fluorinated derivatives should be more resistant to metabolism. Also, we studied the role of the chain length of the thioalkyl substitution and found that an ethyl group was preferable there. Different alkyl and aryl amines were introduced in 4^th^ position of the quinazoline and we observed that only the smallest substitution, methylamine, was good for antituberculosis activity. Compounds (11626245–9) with a primary amine in position 4 were significantly less active. The SAR study further revealed that compounds 11626252 and 11626256 have improved *in vitro* activity against *M*. *tb* H37Rv with MICs of 0.02 and 0.04 μg/mL, respectively. (Fig 1) Interestingly, the parallel introduction of two fluorine atoms in positions 6 and 8 in compound 11826209 did not significantly increase activity. The same level of activity against *M*. *tb* H37Rv *in vitro* was found for the compound 11726148 with 2 fluorine atoms in positions 7 and 8 (MIC 0.05 μg/mL). (Fig 1) Compounds 11626141, 11626142 and 11626252 had no activity against the non-replicating, streptomycin-starved strain 18b, a model for latent TB, and no significant change in the MIC for these compounds was observed when tested against strain H37Rv grown in 7H9-based media containing acetate as the sole carbon source. [7] All four lead compounds had TD_50_ ranging from 9 μg/mL (11626142) to 39 μg/mL (11626252 and 11726148) and medium to high clearance by human microsomes. (Fig 1, S1 Fig) According to the selectivity index, (SI, TD_50_/MIC) 11626141, 11626142, 11726148 and 11626252 have an SI of 575, 100, 780 and 1950, respectively. (Fig 1) Thus, 11626252/11726148 are the most selective compounds from this series and were tested further in additional experiments.

The antitubercular activity of 11626141, 11626142 and 11626252 was also determined *ex vivo* in THP-1 macrophages infected with *M*.*tb* H37Rv. (Fig 1, S1 Fig) IC_50_ (inhibitory concentration 50%) values are between 0.011 to 0.013 μg/mL for the three quinazoline derivatives tested (Fig 1, S1 Fig), indicating potent *ex vivo* activity of these compounds. All three derivatives showed activity against DS- and DR-members of the *M*. *tb* complex (MTBC), as well as against their close relative, *M*. *marinum*. No activity was detected against the other mycobacteria tested nor against a broad range of microorganisms suggesting that these compounds have specific antitubercular activity. (Table 1)

**Table 1:** Susceptibility of different organisms to quinazoline derivatives.

	MIC in μg/mL^a^			
Microorganisms	11626252	11626141	11626142	RIF
***Bacillus subtilis***	≥100	≥100	≥100	0.3
***Candida albicans***	≥100	≥100	44.3	2.2
***Corynebacterium diphtheriae***	≥100	≥100	44.3	0.5
***Corynebacterium glutamicum ATCC13032***	47.2	66.8	75	0.004
***Enterococcus faecalis***	≥100	≥100	≥100	3.9
***Escherichia coli PQ37***	≥100	≥100	≥100	6.5
***Listeria monocytogenes***	≥100	≥100	≥100	0.6
***Micrococcus luteus***	≥100	≥100	≥100	0.2
***Mycobacteroides abscessus subsp*. *abscessus 2005–0524***	≥100	62.4	100	18.1
***Mycobacterium avium ATCC15769***	≥100	≥100	49.1	27.3
***Mycobacteroides abscessus subsp*. *bolletii 1999–0888***	≥100	≥100	≥100	24.8
***Mycobacterium bovis BCG Pasteur***	**1.6**	**0.9**	**0.8**	0.0008
***Mycobacterium canettii STB-L***	**0.03**	**0.05**	**0.2**	0.001
***Mycobacterium marinum Strain M***	**0.007**	**0.1**	**0.8**	0.2
***Mycobacteroides abscessus subsp*. *massiliense 2005–0484***	≥100	≥100	≥100	9.5
***Mycobacterium smegmatis mc*^*2*^*155***	32.3	38.4	75.6	0.9
***Mycobacterium tuberculosis Erdman***	**0.02**	**0.01**	**0.1**	0.001
***Mycobacterium tuberculosis H37Rv***	**0.02**	**0.04**	**0.09**	0.0008
***Mycobacterium tuberculosis HN878***	**0.03**	**0.05**	**0.2**	0.0006
***Mycobacterium tuberculosis MDR 59744***	**0.02**	**0.02**	**0.09**	≥10
***Mycolicibacterium vaccae ATCC 15483***	≥100	≥100	≥100	6.6
***Pseudomonas aeruginosa***	≥100	≥100	≥100	0.2
***Pseudomonas putida***	≥100	≥100	≥100	3
***Salmonella typhimurium***	≥100	≥100	≥100	0.8
***Staphylococcus aureus***	≥100	≥100	≥100	1.4

^a^ Values in bold are ≤ 10 μg/mL

### Quinazoline derivatives target cytochrome *bc*_*1*_

**Fig 2:**
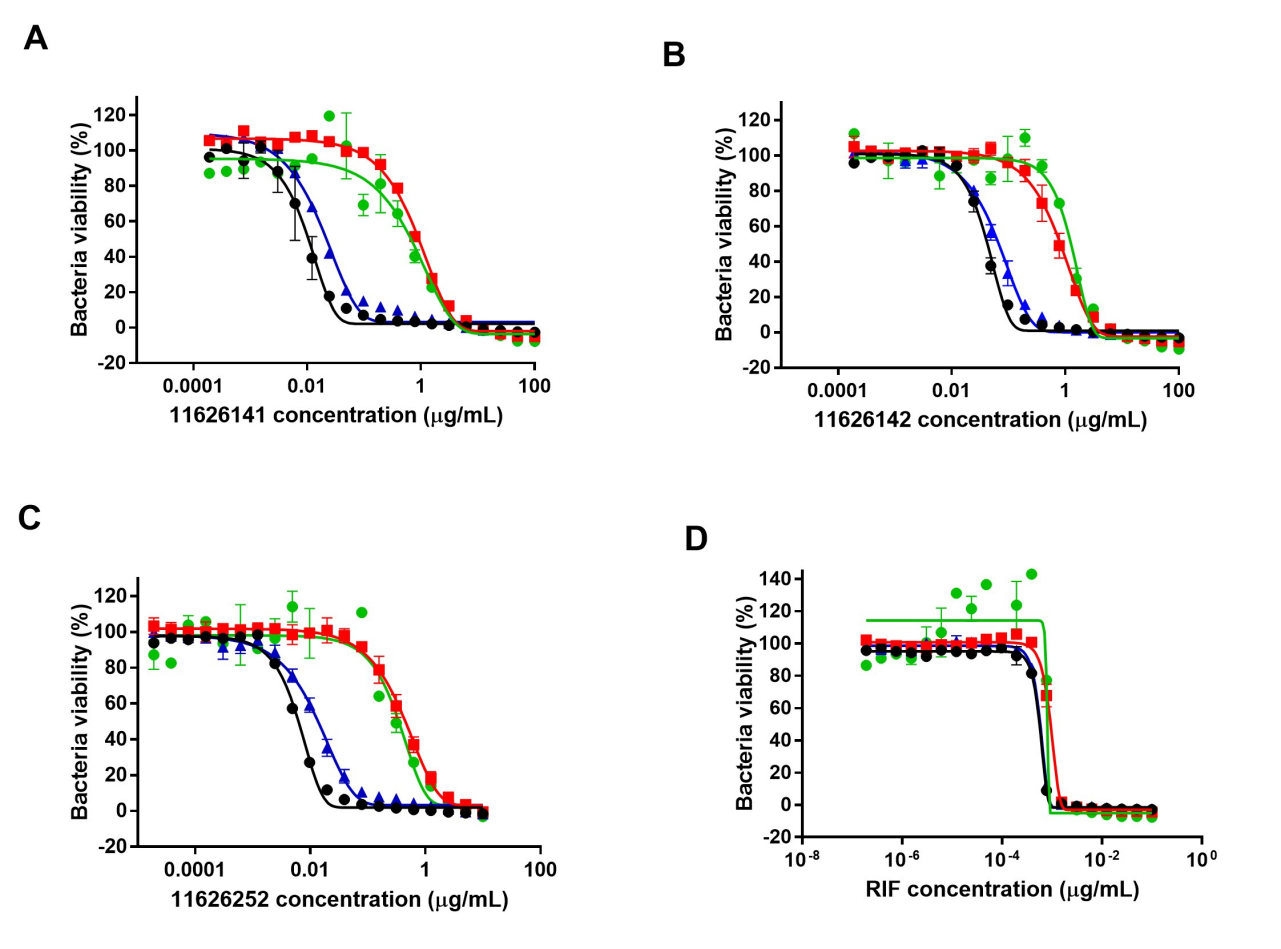
Mutation L356V in QcrA is responsible for quinazoline resistance in *M*. *tb*. Susceptibility to (A) 11626141, (B) 11626142, (C) 11626252 and (D) Rifampicin (RIF) was assessed in strains H37Rv-pYUB412 (black circle), H37Rv-Rv1777(Arg145Ser) (blue triangle), H37Rv-Rv2195(Leu356Val) (red square), and QuinR-M1 (green circle) by REMA. The graph represents the percentage of bacterial viability ± s.d. according to the drug concentrations. The experiment was performed in biological triplicates.

To identify the target of the quinazoline derivatives, resistant mutants of *M*. *tb* H37Rv were selected in the presence of 11626141 and 11626142. Colonies were obtained at 20X MIC on agar plates for 11626141 and 5X MIC for 11626142. Of ten colonies exposed to 11626142, three were resistant to quinazolines when re-tested by REMA. The resistant mutants, QuinR-M1, QuinR-M2 and QuinR-M3, were analyzed by high-throughput sequencing. Whole-genome sequencing (WGS) of QuinR-M2 and QuinR-M3 revealed the presence of the mutations T934G (Trp312Gly) and G523A (Gly175Ser), respectively, in *rv2196*, which codes for the ubiquinol-cytochrome C reductase QcrB. (S2 Table) One non-synonymous mutation was also found in QuinR-M2. (S2 Table) Interestingly, WGS of QuinR-M1 revealed the presence of the transversion T1066G in *rv2195*, which codes for the Rieske iron-sulphur protein QcrA. (S2 Table) A non-synonymous mutation in cytochrome P450 Cyp144 (*rv1777*) was also found in QuinR-M1. To determine if the mutations in QcrA or Cyp144 were involved in quinazoline resistance, H37Rv strains containing the corresponding mutations, T1066G (Leu356Val) in *qcrA* and C433A (Arg145Ser) in *rv1777* were constructed by recombineering. No significant difference (≤ 3-fold change in the MIC) in the susceptibility to 11626141, 11626142 and 11626252 was observed in H37Rv-Rv1777(R145S), compared to the WT strain containing pYUB412. (Fig 2) However, decreased susceptibility was observed in the recombinant mutant H37Rv-Rv2195(L356V), showing that the mutation in QcrA is responsible for resistance to quinazoline derivatives. (Fig 2)

**Fig 3:**
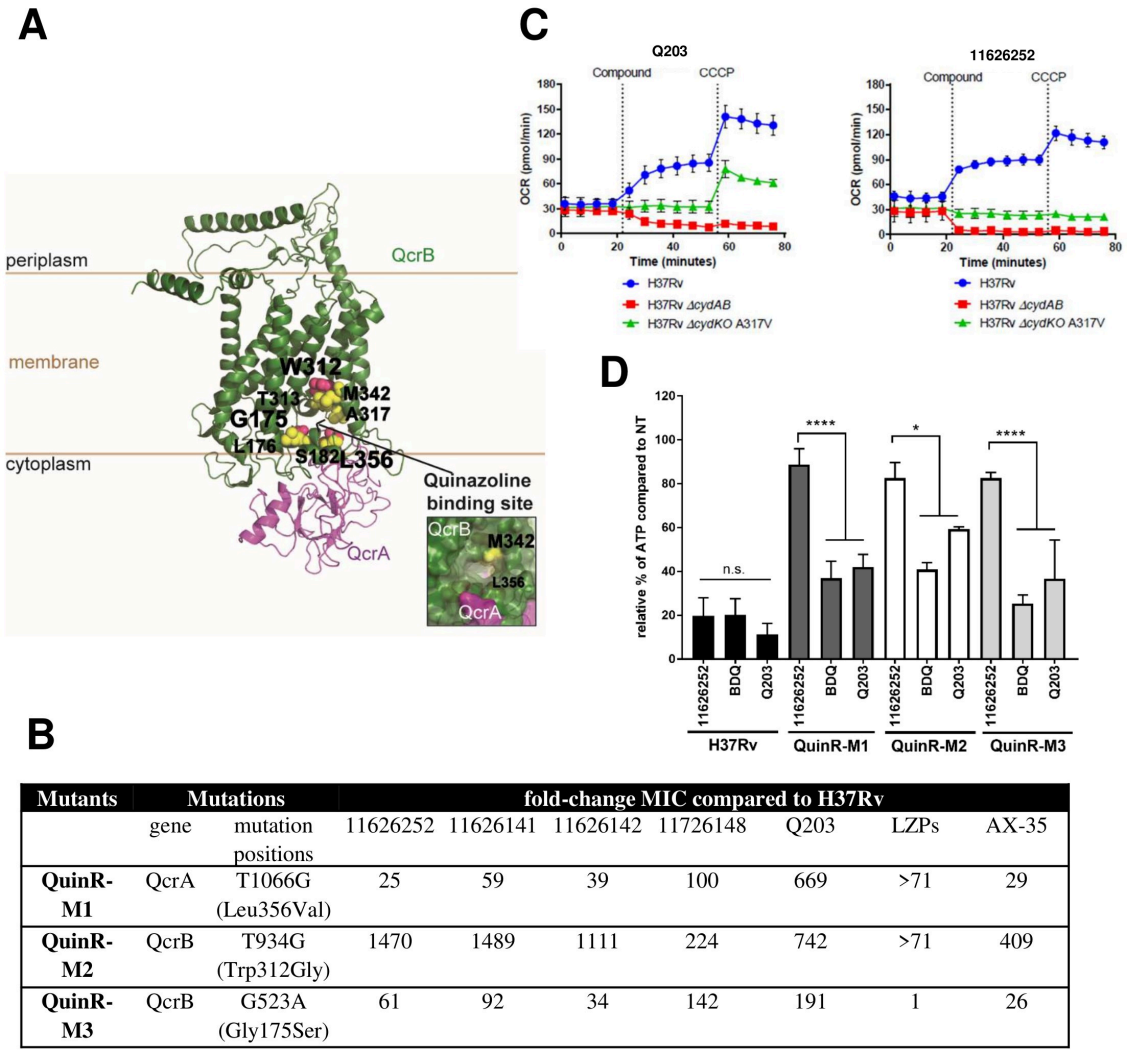
Quinazoline derivatives target cytochrome *bc*_*1*_. (A) QcrA and QcrB of *M*. *tb* were modelled using the homology modelling webserver (Swissmodel) and homology based on chains A and M from the CryoEM structure of the III2IV2 respiratory supercomplex (PDB code 6HWH) from *M*. *smegmatis*. Clustering of mutations associated with resistance to quinazoline derivatives, AX-35, Q203, and LPZs occurs around the quinol oxidation site of QcrB and QcrA. (B) Cross-resistance of QuinR mutants to QcrB inhibitors. The MIC of the quinazoline derivatives, Q203, LPZs and AX-35 was determined and expressed as the fold-change of MIC of each compound compared to *M*. *tb* H37Rv. (C) Bioenergetic analysis of *M*. *tb* upon Q203 and 11626252 treatment followed by the uncoupler CCCP to stimulate maximum respiration. The oxygen consumption rates of wild-type H37Rv, H37Rv Δ*cydAB*, and H37Rv Δ*cyd* KO with a QcrB (A317V) mutation (Q203 resistance SNP) was monitored as described in the Material and methods section. (D) Intracellular ATP levels in H37Rv and quinazoline resistant strains were measured in the absence and presence of BDQ, Q203, and 11626252 at 2.5× MIC after 24 h using BacTiterGlo (Promega). Data from two independent experiments are presented as mean ± SD. Statistical analysis was performed using two-way analysis of variance (ANOVA) with Tukey’s multiple-comparison tests (*, P  < 0.03; ****, P  < 0.0001).

To determine the position of the three mutations identified in the structure of cytochrome *bc*_*1*_ enzyme, models based on chains A and M from CryoEM structure of the respiratory supercomplex III2IV2 from *Mycobacterium smegmatis* (PDB code 6HW6) were made ([8], Fig 3A). As shown in Fig 3A, all the mutations identified in the QuinR mutants map to the quinol oxidation (Qp) site located at the interface of the QcrA and QcrB subunits. [9,10] Cross-resistance to Q203, AX-35 and LPZs was also assessed in the three QuinR mutant strains. (Fig 3B) QuinR-M1 and QuinR-M2 showed a high-level of cross-resistance to all QcrB inhibitors tested. Interestingly, the QuinR-M3 mutant was also cross-resistant to all drugs except LPZs, suggesting that the mutation Gly175Ser did not alter the binding of LPZs to cytochrome *bc*_*1*._ (Fig 3B).

Compounds targeting cytochrome *bc*_*1*_, such as Q203 and AX-35, are known to alter the respiratory profile of *M*. *tb*. [11] Profiling the respiratory response of *M*. *tb* H37Rv to 11626252 treatment was performed as previously described. [11,12] The oxygen consumption rate (OCR) of *M*. *tb* H37Rv increased after exposure to 11626252, similar to that after Q203 addition. (Fig 3C) This increase can be attributed to cytochrome *bd* oxidase, as observed by the rapid and sustained decrease in OCR of a Δ*cydAB* strain of *M*. *tb* H37Rv, even after the addition of the uncoupler carbonyl cyanide m-chlorophenyl hydrazine (CCCP). (Fig 3C) [11–13] However, unlike in the presence of Q203, the *M*.*tb* H37Rv cytochrome *bd* knock-out (KO) strain carrying a QcrB A317V mutation seems to be only partially affected by 11626252. (Fig 3C) The addition of the compound did not cause a deviation in basal respiration in the QcrB A317V mutant strain, as compared to the Q203 response, although no increase in OCR associated with an uncoupled membrane was observed upon CCCP addition after 11626252 treatment. Thus, 11626252 does not affect the basal respiration of the QcrB A317V mutant but does weaken its ability to maintain a favourable membrane potential after CCCP addition.

In addition to altering the respiratory profile of *M*. *tb*, inhibitors of cytochrome *bc*_*1*_ are known to deplete ATP levels. [14–16] The ATP depletion assay was performed on H37Rv and QuinR mutant strains exposed to 11626252 (Fig 3D), with the drugs BDQ and Q203 used as controls. H37Rv cells exposed to 2.5X MIC of 11626252 had a remaining relative ATP level of 19.81±8.125% compared to untreated controls. (Fig 3D) This level is comparable to the depletion observed when the bacteria are treated with BDQ or Q203. (Fig 3D) In all three QuinR mutants, low-level ATP depletion was observed in the presence of 11626252 compared to their respective control backgrounds. Significant ATP depletion was observed for all mutants when exposed to Q203 and BDQ and to a higher level compared to the WT, implying that the mutations identified in QcrA and QcrB alter the activity of the enzyme in the QuinR mutants.

### Transcriptome analysis of *M*. *tb* H37Rv exposed to 11626252

**Fig 4:**
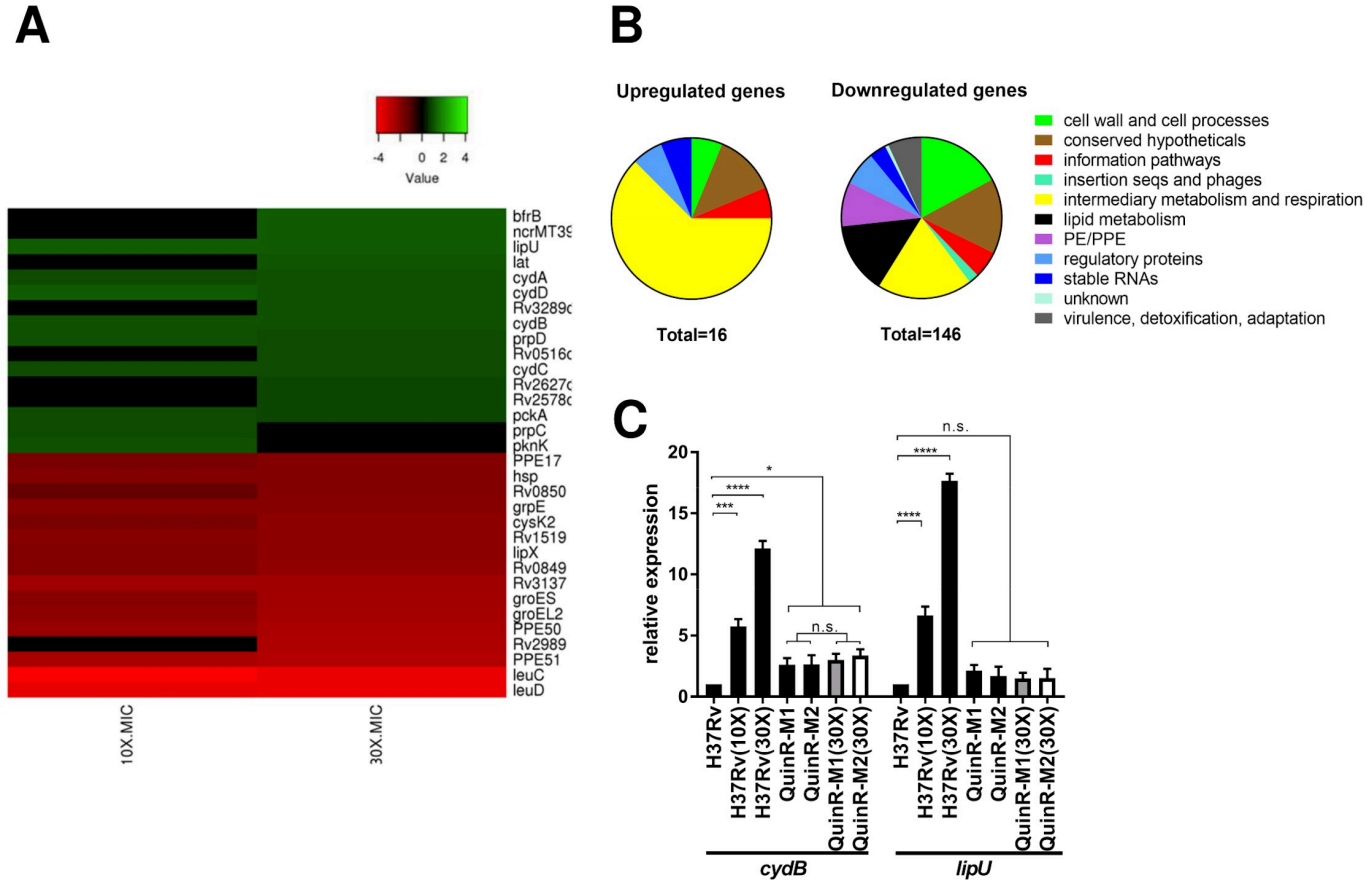
Transcriptome analysis of H37Rv exposed to 11626252. (A) Heat map representing top significantly differentially regulated *M*. *tb* genes (*P*_adj_ ≤ 0.05) after exposure of two independent cultures of *M*. *tb* H37Rv to 11626252 at 10X and 30X MIC for 4 h. The colour scale indicates differential regulation as log2 fold-change of H37Rv with 11626252 treatment relative to H37Rv with vehicle control, DMSO. Upregulation is indicated in red, downregulation is in green, and insignificant log2 fold change values for the condition are in black. Data are from two independent experiments. (B) Global transcriptome response and involvement of different metabolic responses, based on TubercuList classification (https://mycobrowser.epfl.ch/). (C) Fold-change expression of *cydB* and *lipU* determined by qRT-PCR. Bars in black, light grey and white represent the gene expression compared to H37Rv-DMSO, QuinR1-M1 in the presence of DMSO, and QuinR-M2 with DMSO, respectively. Data from two independent cultures are presented as the mean relative expression ± s.d. Statistical analysis was performed using two-way analysis of variance *(*ANOVA) with Tukey’s multiple-comparison tests (*, *P* <0.03; ****, *P* <0.0001).

Transcriptome analysis of *M*. *tb* H37Rv exposed to 11626252 at concentrations of 10X MIC and 30X MIC was performed by RNA-seq. A total of 16 and 146 genes were found to be significantly up- and down-regulated, respectively. (Fig 4A) Most of the up-regulated genes were involved in intermediary metabolism and respiration. (Fig 4B) Overexpression of the cytochrome *bd* oxidase (genes *cydA*, *cydB*, *cydD and cydC*) was observed at both 10X and 30X MIC, indicating compensation by the second terminal oxidase of the electron transport chain (ETC). Genes involved in lipid degradation (*lipU*) and metabolism through the methylcitrate cycle (*prpC* and *prpD*) were also upregulated in the WT H37Rv strain [17,18], suggesting that bacteria exposed to 11626252 may alter their metabolism to utilize fatty-acids through β-oxidation. Overexpression of both *cydB* and *lipU* in *M*. *tb* H37Rv exposed to 11626252 was confirmed by qRT-PCR. (Fig 4C) The expression level of both genes was also measured in the resistant mutants (QuinR-M1 and QuinR-M2) in the presence and absence of 11626252. Interestingly, in the absence of the compound, *cydB* is overexpressed in QuinR-M1 (2.61±0.552 fold change) and QuinR-M2 (2.643±0.751 fold-change), compared to the control strain, *M*. *tb* H37Rv-DMSO. (Fig 4C) No significant difference in *cydB* expression was observed in the resistant mutants exposed to 11626252 (30X MIC) compared to their respective resistant mutants, suggesting that mutations Trp312Gly in QcrB and Leu256Val in QcrA both alter the activity of cytochrome *bc*_*1*_ and induce the expression of cytochrome *bd* oxidase in the quinazoline-resistant mutants. No significant change of *lipU* expression was observed in the QuinR mutants in the presence or absence of the compound.

### 11626252 is bacteriostatic but bactericidal in the absence of *bd* oxidase

**Fig 5:**
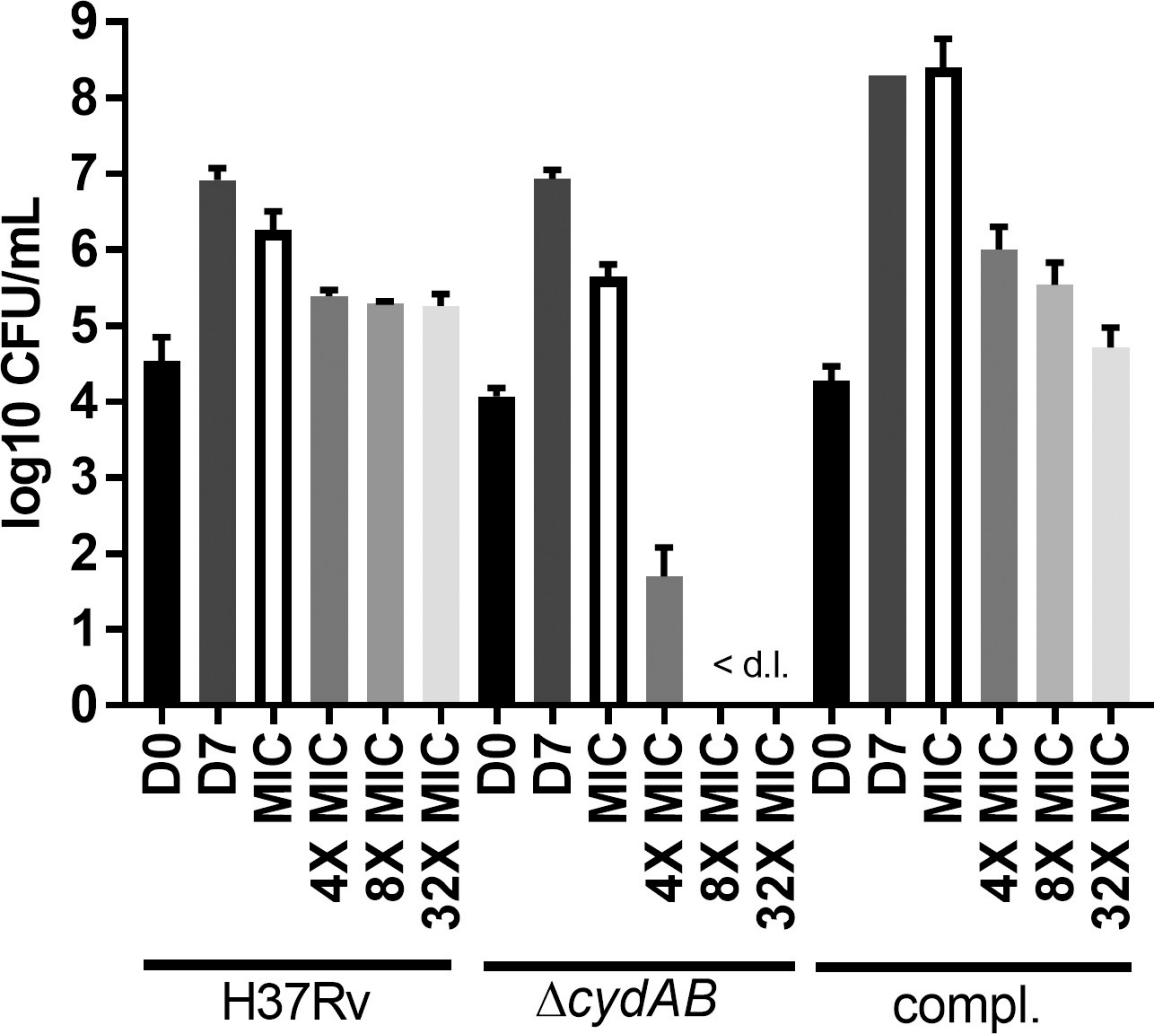
11626252 is bacteriostatic but bactericidal in the absence of the *bd* oxidase. H37Rv, *ΔcydAB* and the complemented strain were exposed to increasing concentrations (1X MIC-32X MIC) of 11626252 for 7 days. Bacteria were plated on 7H10-OADC on day 0 (D0) to determine the initial number of CFU. After 7 days of exposure, untreated bacteria (D7) were plated for CFU enumeration. Results are expressed as the mean log 10 CFU/mL ± s.d.of two independent experiments. <d.l., below limit of detection.

As observed from the transcriptome analysis of bacteria exposed to 11626252, cytochrome *bd* oxidase can compensate for inhibition of cytochrome *bc*_*1*_. CFU enumeration was performed after exposure to increasing concentrations of 11626252 (1X - 32X MIC) in strains *M*. *tb* H37Rv, *M*. *tb* H37Rv Δ*cydAB* and the complemented mutant. [19] 11626252 is a bacteriostatic agent against H37Rv, (Fig 5) however, in the absence of cytochrome *bd* oxidase, *M*. *tb* is killed at a concentration equivalent to 4X MIC, which is consistent with a cidal activity. The bacteriostatic effect is restored in the complemented mutant, indicating that *M*. *tb* only dies *in vitro* in the absence of both terminal oxidases.

### The activity of quinazoline derivatives *in vivo*

**Fig 6:**
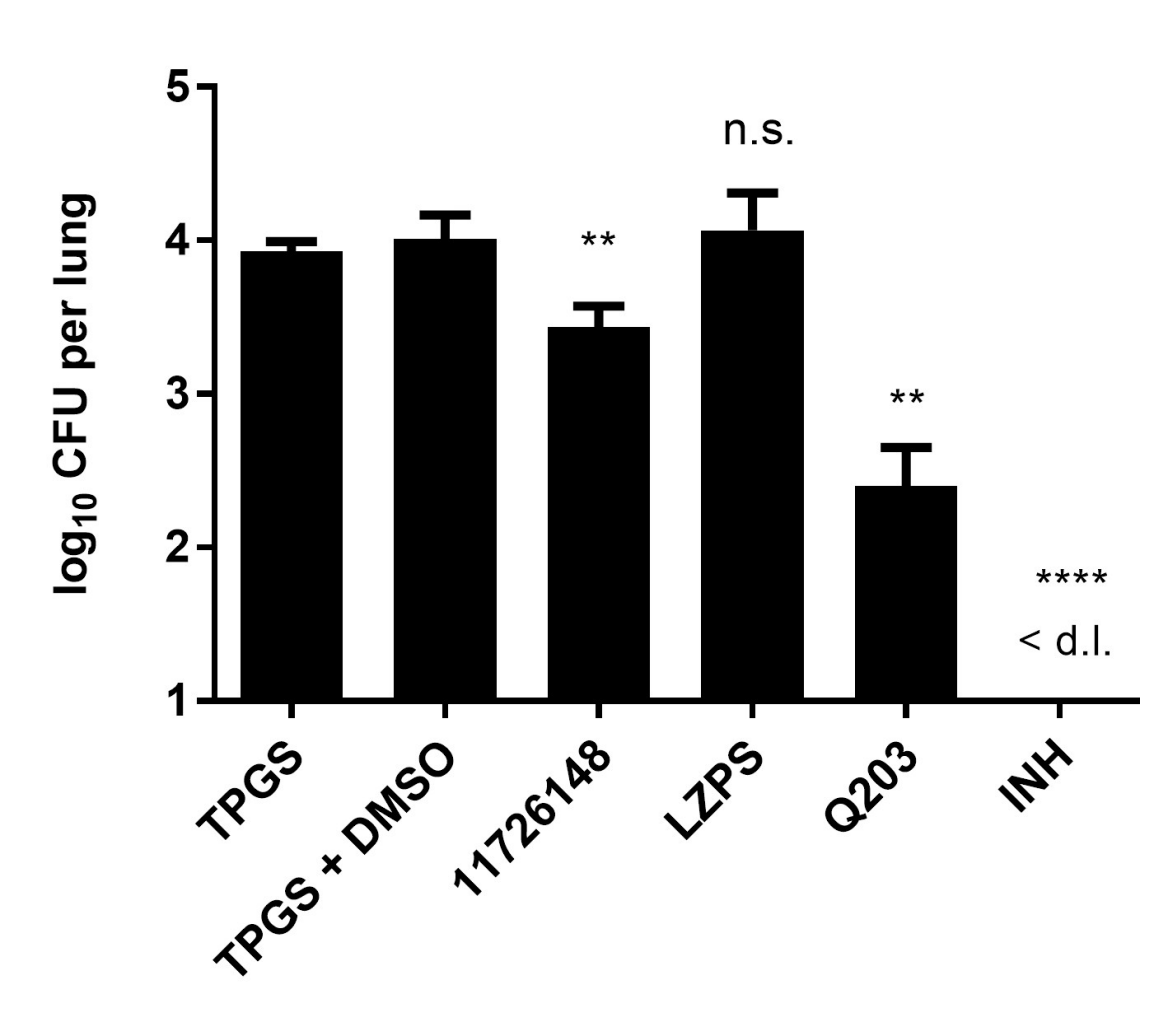
*In vivo* activity of 11726148 in an acute model of murine TB. Compounds were administered by gavage at the following doses: 11726148, 150mg/kg; isoniazid (INH), 25mg/kg; lansoprazole sulfide (LPZS), 300mg/kg and Q203, 25mg/kg. 11726148 and LZPS were prepared in 20% TPGS and Q203 in 20% TPGS+1% DMSO. Bars represent the mean ± s.d. of CFUs from 5 Balb/c mice per group. Significance in difference relative to untreated groups (TPGS and TPGS+DMSO) were calculated using a Student t-test. *P< 0.05; **P< 0.005; ***P> 0.0001.<d.l., below limit of detection.

The activity of selected quinazolines was determined *in vivo* in both the acute and chronic models of murine TB. No activity of 11626252, Q203 and LPZs was observed in the chronic model, as previously described. (S2 Fig) [11] When administered in the acute model at a concentration of 150 mg/kg for 10 days, 11726148 decreased the bacterial burden in the lungs by 0.51 log10 CFU/organ (*p-value* 0.004) compared to the untreated TPGS control (Fig 6).

## Discussion

In this investigation, we characterized the mechanism of action of a new series of quinazoline derivatives active against the MTBC and *M*. *marinum*. (Fig 1 and Table 1) Lead compounds 11626252 and 11726148 have good selectivity against *M*. *tb in vitro* with SIs of 1950 and 780, respectively. (Fig 1) Both compounds result from consistent SAR studies and have very similar chemical structures and properties. Compounds 11626141, 11626142 and 11626252 have similar activity in THP-1 infected macrophages. (Fig 1, S1 Fig) However, 11626252 and 11726148 have both improved stability in human microsomes and reduced HepG2 cytotoxicity compared to 11626141 and 11626142. (Fig 1, S1 Fig) The clearance in human microsomes of compounds 11626252 (Cl_int_ 18.1 μL/min/mg protein) and 11726148 (Cl_int_ 28.7 μL/min/mg protein) is comparable to that of most conventional antitubercular drugs, which have a Cl_int_ in human microsomes lower than 50 μL/min/mg protein. [20] Thus, from the results obtained *in vitro* and *ex vivo*, 11626252 and 11726148 are the best antitubercular compounds in this series. 11726148 decreased the bacterial burden *in vivo* by 0.51 log10 CFU/organ (*p-value* 0.004) in the lungs of mice treated for 10 days. (Fig 6) No activity was observed for 11626252 in the chronic model of murine TB (S2 Fig). This can be readily explained by rerouting of the respiratory pathway toward the less energy-efficient cytochrome *bd* oxidase during the chronic phase of infection in the lungs [21], whereas, during exponential growth, *M*. *tb* utilizes the respiratory pathway terminating in the *aa*_*3*_-type oxidase. The lack of activity in this model could also be due to the dosage used and/or compound metabolism *in vivo*, and further analysis of the pharmacokinetic properties of these compounds would be required to assess their potential as TB drug candidates. As synergism was previously observed with RIF and PZA for the pre-clinical candidate TB-47, a pyrazolopyrimidine which targets cytochrome *bc1*, *in vivo*. [22], it will indeed be of interest for further development to assess the interactions of 2-Ethylthio-4-methylaminoquinazoline derivatives with RIF and PZA.

WGS of the three QuinR mutants revealed that two of them have a mutation in QcrB (Trp312Gly and Gly175Ser) whereas one had a mutation in QcrA (Leu356Val). (S2 Table) QcrA and QcrB are the Rieske iron-sulfur protein and *b* subunit of cytochrome *bc*_*1*_, respectively, and thus are components of the cytochrome *bc*_*1*_*-aa*_*3*_ supercomplex of the ETC. During aerobic respiration, electrons are donated to the menaquinone pool and transferred to oxygen through two branches: the proton pumping cytochrome *bc*_*1*_*-aa*_*3*_ supercomplex and the less energy efficient, but higher-affinity cytochrome *bd* oxidase. [23] Lately, several anti-TB drug candidates were found to target cytochrome *bc*_*1*_*-aa*_*3*_, including the drug candidate Q203. Q203 acts by competing with menaquinone binding at the Qp site of the cytochrome *bc*_*1*_*-aa*_*3*_, also known as the stigmatellin pocket. [15] Mutations causing resistance to QcrB inhibitors (e.g. Q203, LPZs and AX-35) have thus far been found solely in the stigmatellin pocket of QcrB. [11,14,15] According to our model, all QuinR mutations map to this pocket, including the mutation Leu356Val in QcrA (Fig 3A). Based on the Q-cycle model, oxidation of quinol molecules occurs at the interface of cytochrome *b* and the 2Fe–2S cluster domain of the Rieske protein, which forms the catalytic center P. [24,25] Thus, mutations in QcrA can indeed confer resistance to cytochrome *bc*_*1*_ inhibitors if located at this interface. By studying the cross-resistance to the known QcrB inhibitors, AX-35, Q203 and LPZs, we established that residues Trp312 and Gly175 on QcrB and Leu356 on QcrA are involved in binding of most cytochrome *bc*_*1*_ inhibitors to the stigmatellin-binding pocket (Fig 3B), with the exception of Gly175, which does not bind to LPZs.

Quinazoline 11626252 treatment can deplete *M*. *tb* of ATP (Fig 3D) and increase the oxygen consumption rate as part of its mechanism to inhibit bacterial growth. (Fig 3C) As with Q203, this increase in OCR after exposure to 11626252 is due to increased cytochrome *bd* activity as the bacterium attempts to maintain a favourable membrane potential for ATP production after inhibition of cytochrome *bc*_*1*_.[12] Compared to Q203, the OCR profile of the QcrB A317V mutant in the presence of 11626252 suggests that there is sufficient menaquinone binding and electron transfer to sustain basal levels of respiration, but not the higher amounts needed under uncoupled membrane conditions. This indicates that the amino acid substitution A317V in QcrB only partially interferes with the binding of 11626252 to QcrB, thus implying that this quinazoline derivative binds differently than Q203. Transcriptome profiling of the drug confirms that the cytochrome *bd* is indeed overexpressed in the WT strains exposed to 11626252. (Fig 4) Inhibition of cytochrome *bc*_*1*_ was reported to induce this compensatory mechanism in mycobacteria, which accounts for the change between bacteriostatic to bactericidal activity of the drug depending on the cytochrome *bd* oxidase expression level. (Fig 5) [11,19] Such compensation was also observed in the QuinR mutants in the absence of inhibitors, which indicates compensation for the altered function of the cytochrome *bc*_*1*_ due to the presence of the resistance mutations alone. (Fig 4) Furthermore, the activation of the methylcitrate cycle, as deduced from overexpression of the genes *pprC* and *pprD*, suggests a modification of bacterial metabolism towards the propionyl-CoA metabolites and a potential role in *M*. *tb* propionyl-CoA detoxification upon 11626252 treatment. ([26], Fig 4)

From a medicinal chemistry point of view compound, 11726148 looks the more promising scaffold for future derivatization and investigation due to the two fluorine atoms in the phenyl moiety protecting this part of the molecule from potential hydroxylation during metabolism. We are confident that the phenyl moiety can be changed on different heterocycles to confer better pharmacological properties. Furthermore, structure-assisted drug design can be used to produce inhibitors that simultaneously contact QcrA and QcrB thereby alleviating the risk of the emergence of resistance. Such resistance-proofed derivatives could serve as back-ups to Q203, now known as Telacebec, that is currently in phase 2 clinical trials.

## Materials and methods

### Ethics statement

All animal experiments were approved by the office Affaires vétérinaires (1066 Epalinges, Canton Vaud, Switzerland) with the authorization Number 3082 according to the guidelines set by the Service de la consommation et des affaires vétérinaires federal (Canton Vaud, Switzerland).

### Bacterial, cells strains and growth conditions

*M*. *tb* H37Rv was grown in Middlebrook 7H9 media (Difco) supplemented with 10% albumin-dextrose-catalase (ADC), 0.2% glycerol and 0.05% Tween-80 (7H9 complete). *Bacillus subtilis*, *Candida albicans*, *Corynebacterium diphtheriae*, *Corynebacterium glutamicum*, *Escherichia coli*, *Micrococcus luteus*, *Pseudomonas putida*, *Salmonella typhimurium*, and *Staphylococcus aureus* were grown in LB broth. *Enterococcus faecalis*, *Listeria monocytogenes*, and *Pseudomonas aeruginosa* were grown in brain heart infusion (BHI) broth. HepG2 cells (ATCC HB-8065) were grown in DMEM (Gibco) media supplemented with 10% inactivated fetal bovine serum (at 37°C with 5% CO_2_)_._ THP-1 monocytes (ATCC TIB-202) were grown in RPMI medium supplemented with 10% inactivated fetal bovine serum and 1mM sodium pyruvate at 37°C with 5% CO_2_.

### Antibiotic susceptibility

Drugs were tested against *M*. *tb* strain H37Rv using the resazurin reduction microtiter assay (REMA) in 96-well plates as previously described. [27] Briefly, a mid-logarithmic phase culture of H37Rv (OD_600nm_ approx. 0.5) was diluted in 7H9 complete medium to an OD_600nm_ of 0.0001. Bacteria (100 μL) were then dispensed in transparent flat-bottom 96 well plates. Two-fold serial dilutions of each drug (resuspended in DMSO) were then prepared. On each plate, controls without drug and media alone were included. Plates were incubated for 6 days at 37°C before the addition of resazurin (0.025% [wt/vol] to 1/10 of the well volume). After overnight incubation, the fluorescence of the resazurin metabolite, resorufin, was determined with excitation at 560 nm and emission at 590 nm, measured using a TECAN Infinite M200 microplate reader. The minimum inhibitory concentration (MIC_99_, referred to as MIC) was determined using the Gompertz equation with GraphPad Prism software (version 7). All drugs were tested at least in duplicate.

Bactericidal activity of 11626252 was determined against strains H37Rv, H37Rv*ΔcydAB* and the complemented mutant. [19] Bacteria were grown in 7H9 complete media until an OD_600nm_ 0.4–0.8. Strains were diluted to an OD 0.0001 (approximately 3X10^4^ CFU/mL) and exposed to concentrations of 1X, 4X, 8X and 32X MIC of 11626252 for 7 days. Treated bacteria were serially-diluted 10-fold and plated on 7H10 agar containing 0.2% glycerol and 10% oleic acid to determine the number of viable cells. Plates were incubated for four weeks at 37°C. CFUs were enumerated, and the results are presented as log_10_CFU/mL± s.d. The experiment was performed twice.

### Cytotoxicity against HepG2 cells

Human HepG2 cells (ATCC HB-8065, 4000 cells/well) were incubated for 3 days with two-fold serially diluted compounds at 37°C, under an atmosphere of 5% CO_2_. Cell viability was determined by the addition of resazurin (0.0025% w/v) for 5 h at 37°C and the fluorescence intensity measured as in REMA.

### Drug-resistant mutant selection

Resistant mutants to 11626141 and11626142 were isolated on 7H11 agar plates containing 0.2% glycerol and 10% OADC (oleic acid-albumin-dextrose-catalase) with 5X, 10X or 20X MIC of the compound (MIC on plates was 10-fold higher than in liquid media). The plates were incubated at 37°C for four weeks. Colonies were streaked on 7H11-agar plates in the absence of the compound and incubated at 37°C for 4 weeks. Isolated colonies were cultured in 7H9 complete media and resistance to 11626141 or 11626142 was confirmed by REMA.

### Whole-genome sequencing

Genomic DNA was extracted using the QiaAMP UCP Pathogen Minikit (Qiagen) as per the manufacturer’s instructions. Whole-genome sequencing was performed using Illumina technology (HiSeq 2500 instrument) with sequencing libraries prepared using the KAPA HyperPrep kit (Roche). All raw reads were adapter- and quality-trimmed with Trimmomatic v0.33 [28] and mapped onto the *M*. *tb* H37Rv reference genome (RefSeq NC_000962.3) using Bowtie2 v2.2.5. [29] The bamleftalign program from the FreeBayes package v0.9.20–18 [30] was used to left-align indels. Reads with mapping quality <8 and duplicate reads were omitted. Variant calling was done using VarScan v2.3.9 [31] with the following cut-offs: minimum overall coverage of 10 non-duplicated reads, minimum of five non-duplicated reads supporting the SNP, base quality score >15, and a SNP frequency above 30%. The rather low thresholds, especially the SNP frequency, were deliberately chosen to avoid missing potential variants in alignment-difficult regions, or in case of mixed population. All putative variants unique to the mutant strains were manually checked by inspecting the alignments.

### Synthesis of quinazoline derivatives

Most quinazoline derivatives were synthesized in five chemical steps from substituted 2-fluorobenzoic acid or their chloroanhydride which reacted with isothiourea to give N-benzoylimidothiocarbamate. (S1 Table and S3 Fig) These intermediates were heated to close the pyrimidine ring with the formation of the quinazoline-4-one scaffold. This scaffold was introduced into the successive reactions of chlorination by phosphorus (V) oxychloride and the nucleophilic displacement reaction of an active chlorine atom by the corresponding amine. Several compounds bearing phenyl substitutes were synthesized by the Suzuki reaction with phenylboronic acid using tetrakis(triphenylphosphine)palladium as a catalyst. (S1 Table and S3 Fig) All synthesized and tested compounds had purity >97% and were stable in working solutions.

### QcrB and QcrA model

The QcrA and QcrB proteins of *M*. *tb* were modelled using the homology modelling webserver (Swiss model) and the A and M chains from the CryoEM structure of the III2IV2 respiratory supercomplex from *Mycobacterium smegmatis* as template ([8], PDB code 6HWH). Illustrations were made using Pymol version 2.0 software. [32]

### Gene replacement

Recombineering was performed in *M*. *tb* H37Rv containing the plasmid pJV53 as previously described. [14] Briefly, 70-mer oligonucleotides (leading and lagging strands) containing the desired mutations in *rv2195* (*qcrA*) and *rv1777* (Cyp144), (see below) were transformed into an acetamide-induced culture (0.2% v/v; 8h) of *M*. *tb* H37Rv-pJV53. Recombinant mutants were selected using the integrative plasmid pYUB412 [33] on 7H10 agar plates (containing 50μg/mL hygromycin) incubated at 37°C for four weeks. Colonies were screened by PCR for the mutations in *rv2195*and *rv1777* using the primers listed in S3 Table and sent for Sanger sequencing to confirm the presence of the mutation of interest.

### ATP-depletion assay

Log-phase cultures of wild-type H37Rv and quinazoline-resistant mutants (about 10^6^ CFU/mL) were exposed to the test compounds for 24 h in a final volume of 100 μl and incubated with BacTiterGlo Reagent (Promega) (v/v 4:1) for 5 min in the dark. Luminescence was measured on a TECAN Infinite M200 in relative light units (RLU) with an integration time of 1s.

### Transcriptomic analysis and qPCR

Wild-type and quinazoline-resistant H37Rv cultures were grown to mid-log phase and exposed to DMSO (vehicle control) or 11626252 for 4 h at 37°C. Cells were harvested by centrifugation and pellets were stored in 1 ml of TRIzol reagent (Thermo Fisher Scientific) at -80°C until further processed. Cells were lysed by bead-beating and total RNA was extracted by phenol-chloroform with DNAse treatment (RQ1 RNase–free DNase, Promega). Library preparation was done using the Ribo-zero rRNA removal kit (Illumina) for Gram-positive bacteria to deplete rRNA from total RNA. Two biological replicates for each strain were prepared for RNA-seq. Reads were adapter- and quality-trimmed with Trimmomaticv0.3 [28] and mapped onto the *M*. *tb* H37Rv reference genome (RefSeq NC_000962.3) using Bowtie2 v2.2.5 [29]. Counting reads over features was done with featureCounts from the Subread package v1.4.6 [34] and DESeq2 [35] was used to identify differentially expressed genes.

For qPCR, cDNA was prepared from total RNA using a SuperScript III First-strand Synthesis kit (Invitrogen) and analyzed by qPCR, in duplicate, for targeted gene expression using Power SYBR GreenPCR Master Mix (Applied Biosystems) on a QuantStudio 5 Real-Time PCR system (Thermo Fisher Scientific). The house-keeping gene *sigA* was used for normalization and the ΔΔCt method was used for quantification.

### Oxygen consumption rate (OCR) measurement

All strains of *M*. *tb* used were cultured in Middlebrook 7H9 media (Difco) supplemented with 10% OADC (Difco) and 0.01% Tyloxapol (Sigma) at 37 °C, to an OD_600_ ~ 0.6–0.8. *M*. *tb* H37Rv was obtained from BEI Resources (NR-123), *M*. *tb* H37Rv Δ*cydAB* [13] and *M*. *tb* H37Rv Δ*cydKO* A317V [9] were gifts from Dr. Digby Warner and Dr. Helena Boshoff, respectively. *M*. *tb* oxygen consumption rate (OCR) was measured using the XF96 Seahorse XF Analyzer (Agilent) as previously described. [12] In short, *M*. *tb* bacilli were adhered to the bottom of a XF96 cell culture microplate (Agilent) at a density of 2x10^6^ bacilli/well using Cell-Tak cell adhesive (Corning). Extracellular flux analysis was carried out in unbuffered 7H9 media, at pH 7.35, containing 0.2% glucose. Basal OCR was measured for ~19 min before the automatic addition, through the drug ports of the XF96 sensory cartridge (Agilent), of either Q203 (final concentration of 0.3 μM, 100X the MIC_50_) or 11626252 (final concentration of 8.4 μM, 100x the MIC_50_) to the three different *M*. *tb* strains. Q203 was a gift from Dr. Helena Boshoff. The deviations from basal respiration, caused by compound addition, were measured for ~35 min before the addition of the uncoupler CCCP (final concentration of 2 μM, Sigma) to induce maximal OCR, after which OCR was measured for a final ~19 min. OCR data points are representative of the average OCR after 3 min of continuous measurement; the error calculated automatically by the Seahorse Wave Desktop 2.3.0 software (Agilent) from the OCR measurements from at least four replicate wells. OCR plots are representative of two independent experiments performed, and data representation was done using GraphPad Prism 7.

### *Ex vivo* drug susceptibility

Drug susceptibility *ex vivo* was determined in differentiated human THP-1 macrophages (ATCC TIB-202). THP-1 monocytes (1X10^5^ cells/well) were differentiated overnight using 100 ng/mL PMA and seeded in a 96 well-plate. The next day, differentiated macrophages were infected with a mid-logarithmic phase culture of *M*. *tb* H37Rv (OD 0.4–0.8) at an MOI 5. Infection was allowed for 5h. Extracellular bacteria were removed by washing cells twice with pre-warmed (37°C) PBS 1X. RPMI media containing two-fold serial dilutions of compounds was added to the infected cells and incubated for 48h at 37°C with 5% CO_2_. THP-1 viability was determined using PrestoBlue (ThermoFisher). Cells were incubated for 1h in the presence of PrestoBlue and fluorescence monitored by a TECAN F420. The percentage of cell viability was determined by subtracting values for non-treated infected cells from the non-infected cells, and results were plotted using GraphPad Prism version 7. Experiments were performed in two biological replicates.

### *In vivo* drug susceptibility

Balbc/ByJ mice (5–6 weeks old) were infected with a low-dose aerosol (~300 CFU /lung) of *M*. *tb* H37Rv South Africa (kindly provided by Valerie Mizrahi). For the acute model, treatment was initiated 1 day post-infection and administered by gavage daily (in a volume of 200 μl) for 10 days, at the following concentrations: INH, 25 mg/kg; 11726148, 150mg/kg; Q203, 25mg/kg; LZPs, 300mg/kg. 11726148 and LZPs were prepared in 20% D-ɑ-tocopheryl polyethylene glycol succinate (TPGS-Sigma), Q203 was prepared in 20% TPGS containing 1% DMSO (TPGS+DMSO) and INH was prepared in distilled water.

For the chronic model, treatment began 4 weeks after infection and compounds were prepared and administered by gavage 5 days a week for 4 weeks at the following concentrations: 11626252, 100mg/kg; isoniazid (INH), 25mg/kg and Q203, 10mg/kg. 11626252 and Q203 were prepared in 20% TPGS. INH was prepared in distilled water. At the end of the experiments, all mice were sacrificed, and the bacterial load in the lungs and spleen was determined by plating dilutions of organ homogenates on 7H10 agar plates containing 10% OADC, cycloheximide (10 μg/ml), and ampicillin (50 μg/ml). Plates were incubated for 4 weeks at 37°C before CFU enumeration. CFU counts were log10 transformed before analysis as mean log10 CFU ± s.d., and compared using Student's t-tests in Prism version 7.0 (GraphPad).

## Supporting information

S1 Table List of quinazoline derivatives tested. (DOCX)

S2 Table List of mutations identified by WGS of quinazoline mutants. (DOCX)

S3 Table List of strains, plasmids and primers used in this study. (DOCX)

S1 Fig *Ex vivo* activity and drug stability of quinazoline derivatives. (A) THP-1 viability was determined in H37Rv-infected macrophages for 11626141 (black circle), 11626142 (black diamond) and 11626252 (black squares). Rifampicin (RIF; red inverted triangles) was used as a control. (B) Stability of the compounds was determined in human microsomes. Carbamazepine (CBZ) and nifedipine (NIF) were used as controls.
(DOCX)

S2 Fig *In vivo* activity of 11626252 in a chronic model of TB. Drugs were administered by gavage at the following doses: 11626252, 100mg/kg; isoniazid (INH), 25mg/kg and Q203, 10mg/kg. 11626252 and Q203 were prepared in 20% TPGS. INH was prepared in distilled water. Bars represent the mean ± s.d. of CFUs from 5 Balb/c mice per group. Significance in difference relative to NT groups (TPGS) was calculated using a Student t-test. *P< 0.05; **P< 0.005; ***P<0.0001.
(DOCX)

S3 Fig Synthesis of quinazoline derivatives. The general procedure of the quinazoline derivatives synthesis is described in step a) to f).
(DOCX)
