# Supplementary material for: New 2-Ethylthio-4-methylaminoquinazoline derivatives inhibiting two subunits of cytochrome bc1 in Mycobacterium tuberculosis
Source: PLoS Pathog. 2020 Jan 23;16(1):e1008270. doi: 10.1371/journal.ppat.1008270 (PMC6999911; doi:10.1371/journal.ppat.1008270)
Supplement: S1 Table — (DOCX) [file ppat.1008270.s001.docx]

## Table S1: List of quinazoline derivatives tested

| **Compound** | **R^1^** | **R^2^** | **R^3^** | **R^4^** | **R^5^** | **R^6^** | **R^7^** |
| --- | --- | --- | --- | --- | --- | --- | --- |
| 11626134 | H | H | H | H | Me | H | Me |
| 11626135 | H | H | H | H | Me | Me | Me |
| 11626136 | H | H | H | H | Me | H | Et |
| 11626137 | H | H | H | H | Me | H | *i*Pr |
| 11626138 | H | H | H | H | Me | H | CH_2_Ph |
| 11626140 | H | H | H | H | Me | H | Ph |
| 11626141 | H | H | H | H | Et | H | Me |
| 11626142 | H | H | H | H | Et | H | Et |
| 11626143 | H | H | H | H | Et | H | CH_2_Ph |
| 11626145 | H | H | H | H | Et | Me | Me |
| 11626146 | H | H | H | H | Et | H | *i*Pr |
| 11626147 | H | H | H | H | Et | H | Ph |
| 11626164 | H | H | H | F | Me | H | Me |
| 11626165 | H | H | H | F | Me | H | Et |
| 11626167 | H | H | H | F | Me | H | *i*Pr |
| 11626168 | H | H | H | F | Me | H | Ph |
| 11626244 | H | H | H | F | Et | H | Me |
| 11626245 | H | H | H | F | Me | Me | Me |
| 11626246 | H | H | H | F | Me | H | H |
| 11626247 | H | H | H | H | Me | H | H |
| 11626248 | H | H | H | F | Et | H | H |
| 11626249 | H | H | H | H | Et | H | H |
| 11626250 | H | H | H | F | CH_2_Ph | H | Me |
| 11626251 | H | H | H | F | CH_2_Ph | H | Et |
| 11626252 | F | H | H | H | Et | H | Me |
| 11626253 | F | H | H | H | Et | H | Et |
| 11626254 | H | F | H | H | Et | H | Me |
| 11626255 | H | F | H | H | Et | H | Et |
| 11626256 | H | H | F | H | Et | H | Me |
| 11626257 | H | H | F | H | Et | H | Et |
| 11626258 | H | H | NO_2_ | H | Et | H | Me |
| 11626259 | H | H | NO_2_ | H | Et | H | Et |
| 11726001 | H | H | CF_3_ | H | Et | H | Me |
| 11727002 | H | H | CF_3_ | H | Et | H | Et |
| 11726003 | H | H | H | H | Pr | H | Me |
| 11726004 | H | H | H | H | Pr | H | Et |
| 11726027 | CF_3_ | H | H | H | Et | H | Me |
| 11727028 | CF_3_ | H | H | H | Et | H | Et |
| 11726029 | F | H | H | H | Pr | H | Me |
| 11726030 | F | H | H | H | Pr | H | Et |
| 11726037 | H | H | H | H | *i*Pr | H | Me |
| 11726038 | H | H | H | H | *i*Pr | H | Et |
| 11726039 | F | H | H | H | *i*Pr | H | Me |
| 11726040 | F | H | H | H | *i*Pr | H | Et |
| 11726077 | H | H | NH_2_ | H | Et | H | Me |
| 11726078 | H | H | NHAc | H | Et | H | Me |
| 11726080 | NO_2_ | H | NO_2_ | H | Et | H | Me |
| 11726142 | H | F | F | H | Et | H | Me |
| 11726146 | F | F | F | H | Et | H | Me |
| 11726147 | Cl | H | H | H | Et | H | Me |
| 11726148 | F | F | H | H | Et | H | Me |
| 11826051 | H | H | Br | H | Et | H | Me |
| 11826052 | H | H | Me | H | Et | H | Me |
| 11826058 | H | CF_3_ | H | H | Et | H | Me |
| 11826059 | Me | H | H | H | Et | H | Me |
| 11826060 | OEt | F | H | H | Et | H | Me |
| 11826061 | H | Br | H | H | Et | H | Me |
| 11826062 | H | CN | H | H | Et | H | Me |
| 11826063 | OMe | Cl | H | H | Et | H | Me |
| 11826064 | H | Cl | H | H | Et | H | Me |
| 11826065 | F | Me | H | H | Et | H | Me |
| 11826066 | Cl | F | F | H | Et | H | Me |
| 11826070 | H | OCH_2_Ph | H | H | Et | H | Me |
| 11826067 | Cl | F | H | H | Et | H | Me |
| 11826209 | F | H | F | H | Et | H | Me |
| 11826053 | H | Ph |  |  |  |  |  |
| 11826054 | H | 4-FPh |  |  |  |  |  |
| 11826055 | H | 3,4-F,FPh |  |  |  |  |  |
| 11826056 | H | 4-CF_3_Ph |  |  |  |  |  |
| 11826057 | H | 2,4-F,FPh |  |  |  |  |  |
| 11826068 | Ph | H |  |  |  |  |  |
| 11826069 | 4-FPh | H |  |  |  |  |  |
| 11826071 | 3,4-F,FPh | H |  |  |  |  |  |
| 11826087 | 4-CF_3_Ph | H |  |  |  |  |  |
| 11826088 | 2,4-F,FPh | H |  |  |  |  |  |
