# Supplementary material for: New 2-Ethylthio-4-methylaminoquinazoline derivatives inhibiting two subunits of cytochrome bc1 in Mycobacterium tuberculosis
Source: PLoS Pathog. 2020 Jan 23;16(1):e1008270. doi: 10.1371/journal.ppat.1008270 (PMC6999911; doi:10.1371/journal.ppat.1008270)
Supplement: S2 Table — (DOCX) [file ppat.1008270.s002.docx]

## Table S2: List of mutations identified by WGS of quinazoline mutants

| Gene | Gene annotation | QuinR-M1 | QuinR-M2 | QuinR-M3 |
| --- | --- | --- | --- | --- |
| *rv1777* | Probable cytochrome P450 144 Cyp144 | C433A  (Arg145Ser) |  |  |
| *rv2195* | Probable rieskeiron-sulfurproteinQcrA | T1066G  (Leu356Val) |  |  |
| *rv2196* | Probable ubiquinol-cytochrome C reductase QcrB (cytochrome B subunit) |  | T934G  (Trp312Gly) | G523A  (Gly175Ser) |
| *rv2305* | Unknown protein |  | C789A  (Asn263Lys) |  |
