# Supplementary material for: New 2-Ethylthio-4-methylaminoquinazoline derivatives inhibiting two subunits of cytochrome bc1 in Mycobacterium tuberculosis
Source: PLoS Pathog. 2020 Jan 23;16(1):e1008270. doi: 10.1371/journal.ppat.1008270 (PMC6999911; doi:10.1371/journal.ppat.1008270)
Supplement: S3 Table — (DOCX) [file ppat.1008270.s003.docx]

## Table S3 List of strains, plasmids and primers used in this study

| Strains | Description | Reference |
| --- | --- | --- |
| H37Rv | ATCC® 27294™ | [1] |
| H37Rv (South Africa) | ATCC® 27294™, kindly provided by Valerie Mizrahi | [2] |
| H37Rv-pJV53 | *M. tuberculosis* H37Rv containing the plasmid pJV53 (Kan^R^) | [3] |
| QuinR_M1 | Quinazoline resistant mutant M1. Background *M. tuberculosis* H37Rv | This study |
| QuinR_M2 | Quinazoline resistant mutant M2. Background *M. tuberculosis* H37Rv | This study |
| QuinR_M3 | Quinazoline resistant mutant M3. Background *M. tuberculosis* H37Rv | This study |
| H37Rv-Rv2195(L356V) | H37Rv-pJV53containing the mutation T1066G (Leu356Val) in Rv2195 and pYUB412 (Hygro^R^). | This study |
| H37Rv-Rv1777(R145S) | H37Rv-pJV53 containingthe mutation C433A (Arg145Ser) in Rv1777, pYUB412 (Hygro^R^) | This study |
| Primers^a^ | **Sequence (5’-3’)** | **Reference** |
| Rv2195-F | GACGAAGCGATGAGGAGGAG | This study |
| Rv2195-R | ACGGGCTCGACAAAGTCACC | This study |
| Rv1777-F | GCGCAGTTCTCGACTTGCAG | This study |
| Rv1777-R | ATCGAGCAGCAGACGCAAGA | This study |
| cydB-F | GACGATGCCTACCGATTCGC | [4] |
| cydB-R | CCAGCCACGTCCAGTCTTTG | [4] |
| lipU-F | CAAAGGAACACAAGCAGGCG | [4] |
| lipU-R | GTCTACCTGGTTCCTCGCTG | [4] |
| Rv2195-F  (recombineering) | CGAATTCTTCGCGTTCACCAAGGTCTGCTCTCAT**G**TGGGTTGCCCGTCATCGCTGTACGAGCAGCAGAGC | This study |
| Rv2195-R  (recombineering) | GCTCTGCTGCTCGTACAGCGATGACGGGCAACCCA**C**ATGAGAGCAGACCTTGGTGAACGCGAAGAATTCG | This study |
| Rv1777-F  (recombineering) | GCTCGTGCTGCGTCACTTGGCGGCCAAGCGGATC**A**GCGTTATGGAGCAGTTCACCGTACAGGCTGCCGAC | This study |
| Rv1777-R  (recombineering) | GTCGGCAGCCTGTACGGTGAACTGCTCCATAACGC**T**GATCCGCTTGGCCGCCAAGTGACGCAGCACGAGC | This study |

^a^ Primer sequences are 5’-3’. Nucleic acids in bold represent the mutation introduced by recombineering.

References

1. Cole ST, Brosch R, Parkhill J, Garnier T, Churcher C, Harris D, et al. Deciphering the biology of Mycobacterium tuberculosis from the complete genome sequence. Nature. 1998;393: 537–544. doi:10.1038/31159

2. Ioerger TR, Feng Y, Ganesula K, Chen X, Dobos KM, Fortune S, et al. Variation among Genome Sequences of H37Rv Strains of Mycobacterium tuberculosis from Multiple Laboratories. J Bacteriol. 2010;192: 3645–3653. doi:10.1128/JB.00166-10

3. van Kessel JC, Hatfull GF. Recombineering in *Mycobacterium tuberculosis*. Nat Methods. 2007;4: 147–152. doi:10.1038/nmeth996

4. Foo CS, Lupien A, Kienle M, Vocat A, Benjak A, Sommer R, et al. Arylvinylpiperazine Amides, a New Class of Potent Inhibitors Targeting QcrB of Mycobacterium tuberculosis. mBio. 2018;9: e01276-18. doi:10.1128/mBio.01276-18
