# Supplementary material for: New 2-Ethylthio-4-methylaminoquinazoline derivatives inhibiting two subunits of cytochrome bc1 in Mycobacterium tuberculosis
Source: PLoS Pathog. 2020 Jan 23;16(1):e1008270. doi: 10.1371/journal.ppat.1008270 (PMC6999911; doi:10.1371/journal.ppat.1008270)
Supplement: S1 Fig — (A) THP-1 viability was determined in H37Rv-infected macrophages for 11626141 (black circle), 11626142 (black diamond) and 11626252 (black squares). Rifampicin (RIF; red inverted triangles) was used as a control. (B) Stability of the compounds was determined in human microsomes. Carbamazepine (CBZ) and nifedipine (NIF) were used as controls. (DOCX) [file ppat.1008270.s004.docx]

## Figure S1 *Ex vivo* activity and drug stability of quinazoline derivatives

(A) THP-1 viability was determined in H37Rv infected macrophages for **11626141** (black circle), **11626142** (black diamond) and **11626252** (black squares). Rifampicin (RIF; red inverted triangles) was used as a control. (B) Stability of the compounds was determined in human microsomes. Carbamazepine (CBZ) and nifedipine (NIF) were used as controls.

**A**

**B**
