# Supplementary material for: New 2-Ethylthio-4-methylaminoquinazoline derivatives inhibiting two subunits of cytochrome bc1 in Mycobacterium tuberculosis
Source: PLoS Pathog. 2020 Jan 23;16(1):e1008270. doi: 10.1371/journal.ppat.1008270 (PMC6999911; doi:10.1371/journal.ppat.1008270)
Supplement: S2 Fig — Drugs were administered by gavage at the following doses: 11626252, 100mg/kg; isoniazid (INH), 25mg/kg and Q203, 10mg/kg. 11626252 and Q203 were prepared in 20% TPGS. INH was prepared in distilled water. Bars represent the mean ± s.d. of CFUs from 5 Balb/c mice per group. Significance in difference relative to NT groups (TPGS) was calculated using a Student t-test. *P< 0.05; **P< 0.005; ***P<0.0001. (DOCX) [file ppat.1008270.s005.docx]

## Figure S2: *In vivo* activity of 11626252 in a chronic model of TB. Drugs were administered by gavage at the following doses: 11626252, 100mg/kg; isoniazid (INH), 25mg/kg and Q203, 10mg/kg. 11626252 and Q203 were prepared in 20% TPGS. INH was prepared in distilled water. Bars represent the mean ± s.d. of CFUs from 5 Balb/c mice per group. Significance in difference relative to NT groups (TPGS) was calculated using a Student t-test. **P*< 0.05; ***P*< 0.005; ****P*< 0.0001.
