# Supplementary material for: New 2-Ethylthio-4-methylaminoquinazoline derivatives inhibiting two subunits of cytochrome bc1 in Mycobacterium tuberculosis
Source: PLoS Pathog. 2020 Jan 23;16(1):e1008270. doi: 10.1371/journal.ppat.1008270 (PMC6999911; doi:10.1371/journal.ppat.1008270)
Supplement: S3 Fig — The general procedure of the quinazoline derivatives synthesis is described in step a) to f). (DOCX) [file ppat.1008270.s006.docx]

## Figure S3: Synthesis of quinazoline derivatives. The general procedure of the quinazoline derivatives synthesis is described in step a) to f).

**Step a).** A suspension of benzoic acid (6.5 mmol) in 10 mL of carbon tetrachloride was treated by 13,8 mmol of thionyl chloride with 2 drops of dry DMF. The reaction mass was refluxed for 3h, evaporated in a vacuum, and the resultant benzoyl chloride was used in the next step without additional purification.

**Step b).** A suspension of isothiourea (19,6 mmol) in 28 ml of dichloromethane was treated by 5.4 ml (39,1 mmol) triethylamine. The solution obtained was cooled to 0 ^о^С and treated dropwise by corresponding benzoyl chloride (17 mmol) in 2 ml of dichloromethane for 1 h at 0^о^С to -5 ^о^С. After full addition, the reaction mixture was held for 15 min at room temperature, evaporated in vacuum and the oily residue treated with water. The sediment of N-benzoyl imidothiocarbamate was filtered off, washed with water and recrystallized from mixture hexane/ethyl acetate.

**Step c).** A solution of 4,7 mmol N-benzoyl imidothiocarbamatein 10 mL of dry DMF was refluxed for 2h, the reaction mixture was cooled and then treated by crushed ice. The formed precipitate of quinazoline-4-one was filtered off, washed with water and recrystallized from a mixture of hexane/ethyl acetate.

**Step d).** A solution of 2,6 mmol quinazoline-4-one in 4 ml phosphorus (V) oxychloride was treated by 10 mg of triethylamine hydrochloride and refluxed for 2 h. The dark reaction mixture was cooled to room temperature and treated by crushed ice. The resulting oil gradually crystallizes, the precipitate of 4-chloro-2-(methylthio)quinazoline is filtered off, good washed with water until pH=7, dried and recrystallized from hexane.

**Step e).** A suspension of 4-chloro-2-(methylthio) quinazoline (0.89 mmol) in 2 ml of ethanol was treated by 2 ml of 70% ethylamine solution in water. The reaction mass was stirred for 4 hat 40 ^o^C, evaporated in a vacuum, the residue was treated with water and filtered off. Aim 2-(ethylthio)-N-methylquinazolin-4-amine was recrystallized from a mixture of hexane/ethyl acetate.

**Step f**). A solution of 6(7)-bromo-2-(ethylthio)-N-methylquinazolin-4-amine (0.34 mmol) in 5 ml of 1,4-dioxane was treated by 0,36 mmol of corresponding phenylboronic acid,tetrakis(triphenylphosphine)palladium(0) (15%) and solution of 0.18 g (1.7 mmol) of sodium carbonate in 2 ml of water. The reaction mixture was refluxed for 2 h, cooled to room temperature, diluted with cold water and extracted with ethyl acetate (3 x 30 ml). Organic phases were combined, washed by 1N sodium hydroxide solution, water and dried by sodium sulfate. Final 2-(ethylthio)-*N*-methyl-6(7)-phenylquinazolin-4-amine was recrystallized from ethanol.

*N-methyl-2-(methylthio)quinazolin-4-amine*(11626134) Yeild 21%. Mp. 155-7ºС (Hexane /EtOAc).Mass (EI), m/z (I*_relat_*.(%)): 205.2806 [M]^+^ (42).C_10_H_11_N_3_S. ^1^H NMR (DMSO-d_6_ with 0.05% v/v TMS, 300 MHz,): δ 2.52 (3H, s, SCH_3_), 3.00 (3H, d, *J =* 4.5, NCH_3_), 7.36 (1H, ddd, *J =* 1.2, 7.0, 8.2, H6), 7.52 (1H, dd, *J =* 1.2, 8.3, H8), 7.68 (1H, ddd, *J =* 1.4, 7.0, 8.3, H7), 8.08 (1H, dd, *J =* 1.4, 8.2, H5), 8.26 (1H, br s, NH) ppm.

*N,N-dimethyl-2-(methylthio)quinazolin-4-amine*(11626135)Yeild 37%. Mp. 110-2ºС (Hexane). Mass (EI), m/z (I*_relat_*.(%)): 219.3071 [M]^+^ (93). C_11_H_13_N_3_S. ^1^H NMR (DMSO-d_6_ with 0.05% v/v TMS, 300 MHz,): δ 2.52 (3H, s, SCH_3_), 3.32 (6H, s, NCH_3_), 7.34 (1H, t, *J =* 8.2, H6), 7.57 (1H, d, *J =* 8.2, H8), 7.69 (1H, t, *J =* 8.2, H7), 8.09 (1H, d, *J =* 8.2, H5) ppm.

*N-ethyl-2-(methylthio)quinazolin-4-amine*(11626136)Yeild 20%. Mp. 161-4ºС (Hexane /EtOAc). Mass (EI), m/z (I*_relat_*.(%)): 219.3071 [M]^+^ (73). C_11_H_13_N_3_S. ^1^H NMR (DMSO-d_6_ with 0.05% v/v TMS, 300 MHz,): δ 1.23 (3H, t, *J =* 6.9, CH_3_CH_2_), 2.51 (3H, s, SCH_3_), 3.54 (2H, dq, *J =* 5.3, 6.9, CH_3_CH_2_), 7.36 (1H, ddd, *J =* 1.2, 7.0, 8.3, H6), 7.51 (1H, dd, *J =* 1,2, 8.4, H8), 7.68 (1H, ddd, *J =* 1.4, 7.0, 8.4, H7), 8.13 (1H, dd, *J =* 1.4, 8.3, H5), 8.24 (1H, br s, NH) ppm.

*N-isopropyl-2-(methylthio)quinazolin-4-amine* (11626137)Yeild 65%. Mp. 138-141ºС (Hexane). Mass (EI), m/z (I*_relat_*.(%)): 233.3337 [M]^+^(67). C_12_H_15_N_3_S. ^1^H NMR (DMSO-d_6_ with 0.05% v/v TMS, 300 MHz,): δ 1.26 (6H, d, *J =* 6.5, (CH_3_)_2_CH), 2.50 (3H, s, SCH_3_), 4.46 (H, oktet, *J =*  6.5, (CH_3_)_2_CH), 7.36 (1H, ddd, *J =* 1.2, 7.0, 8.2, H6), 7.51 (1H, dd, *J =* 1.2, 8.4, H8), 7.68 (1H, ddd, *J =* 1.4, 8.4, H7), 7.94 (1H, br d, *J =* 6.8, NH), 8.22 (1H, dd, *J =* 1.4, 8.2, H5) ppm.

*N-benzyl-2-(methylthio)quinazolin-4-amine* (11626138) Yeild 30%. Mp. 144-7ºС (Hexane /EtOAc). Mass (EI), m/z (I*_relat_*.(%)): 281.3765 [M]^+^(73). C_16_H_15_N_3_S. ^1^H NMR (DMSO-d_6_ with 0.05% v/v TMS, 300 MHz,): δ 2.46 (3H, s, SCH_3_), 4.75 (2H, d, *J =* 6.50, CH_2_Ph), 7.27 (1H, t, *J =* 7.3, H4(Ph)), 7.28-7.42 (4H, m, Ph), 7.37 (1H, t, *J =* 8.0, H6), 7.53 (1H, d, *J =* 8.0, H8), 7.70 (1H, t, *J =* 8.4, H7), 8.21 (1H, d, *J =* 8.4, H5), 8.82 (1H, br s, NH) ppm.

*2-(methylthio)-N-phenylquinazolin-4-amine* (11626140) Yeild 7%. Mp. 172-4ºС (Hexane /EtOAc). Mass (EI), m/z (I*_relat_*.(%)): 267.3499 [M]^+^(92). C_15_H_13_N_3_S. ^1^H NMR (DMSO-d_6_ with 0.05% v/v TMS, 300 MHz,): δ 2.52 (3H, s, SCH_3_), 7.15 (1H, t, *J =* 7.3, H4(Ph)), 7.41 (2H, t, *J =* 7.3, m-Ph), 7.50 (1H, t, *J =* 8.2, H6), 7.63 (1H, d, *J =* 8.2, H8), 7.79 (1H, t, *J =* 8.4, H7), 7.83 (2H, d, *J =* 7.3, o-Ph), 8.48 (1H, d, *J =* 8.2, H5), 9.82 (1H, br s, NH) ppm.

*2-(ethylthio)-N-methylquinazolin-4-amine* (11626141) Yeild 61%. Mp. 160-3ºС (Hexane /EtOAc). Mass (EI), m/z (I*_relat_*.(%)): 219.3071 [M]^+^ (63). C_11_H_13_N_3_S. ^1^H NMR (DMSO-d_6_ with 0.05% v/v TMS, 300 MHz,): δ 1.35 (3H, t, *J =* 7.3, CH_3_CH_2_), 2.99 (3H, d, *J =* 4.5, NCH_3_), 3.12 (2H, q, *J =* 7.3, CH_3_CH_2_), 7.37 (1H, ddd, *J =* 1.2, 7.0, 8.3, H6), 7.51 (1H, dd, *J =* 1,2, 8.4, H8), 7.68 (1H, ddd, *J =* 1.4, 7.0, 8.4, H7), 8.09 (1H, dd, *J =* 1.4, 8.3, H5), 8.32 (1H, br q, *J =* 4.5, NH) ppm.

*N-ethyl-2-(ethylthio)quinazolin-4-amine* (11626142) Yeild 63%. Mp. 133-7ºС (Hexane /EtOAc). Mass (EI), m/z (I*_relat_*.(%)): 233.3337 [M]^+^ (76). C_12_H_15_N_3_S. ^1^H NMR (DMSO-d_6_ with 0.05% v/v TMS, 300 MHz,): δ 1.23 (3H, t, *J =* 7.1, CH_3_CH_2_N), 1.34 (3H, t, *J =* 7.1, CH_3_CH_2_S), 3.11 (2H, q, *J =* 7.1, CH_3_CH_2_S), 3.54 (2H, dq, *J =* 5.4, 7.1, CH_3_CH_2_N), 7.36 (1H, ddd, *J =* 1.2, 7.0, 8.3, H6), 7.50 (1H, dd, *J =* 1,2, 8.4, H8), 7.68 (1H, ddd, *J =* 1.4, 7.0, 8.4, H7), 8.13 (1H, dd, *J =* 1.4, 8.3, H5), 8.30 (1H, br t, *J =* 5.4, NH) ppm.

*N-benzyl-2-(ethylthio)quinazolin-4-amine* (11626143)Yeild 38%. Mp. 120-4ºС (H_2_O/*i*-PrOH). Mass (EI), m/z (I*_relat_*.(%)): 295.4031 [M]^+^(43). C_17_H_17_N_3_S. ^1^H NMR (DMSO-d_6_ with 0.05% v/v TMS, 300 MHz,): δ 1.24 (3H, t, *J =* 7.3, CH_3_CH_2_), 3.04 (2H, q, *J =* 7.3, CH_3_CH_2_), 4.76 (2H, d, *J =* 5.8, CH_2_Ph), 7.18-7.46 (6H, m, Ph, H6), 7.52 (1H, d, *J =* 8.3, H8), 7.71 (1H, dd, *J =* 7.0, 8.2, H7), 8.22 (1H, d, *J =* 8.2, H5), 8.92 (1H, br t, *J =* 5.8, NH) ppm.

*2-(ethylthio)-N,N-dimethylquinazolin-4-amine* (11626145) Yeild 41%. Mp. 60-2ºС (Hexane). Mass (EI), m/z (I*_relat_*.(%)): 233.3337 [M]^+^(56). C_12_H_15_N_3_S. ^1^H NMR (DMSO-d_6_ with 0.05% v/v TMS, 300 MHz,): δ 1.34 (3H, t, *J =* 7.1, CH_3_CH_2_S), 3.12 (2H, q, *J =* 7.1, CH_3_CH_2_S), 3.32 (6H, s, N(CH_3_)_2_), 7.34 (1H, ddd, *J =* 1.2, 7.0, 8.3, H6), 7.56 (1H, dd, *J =* 1,2, 8.4, H8), 7.69 (1H, ddd, *J =* 1.2, 7.0, 8.4, H7), 8.08 (1H, dd, *J =* 1.2, 8.3, H5) ppm.

*2-(ethylthio)-N-isopropylquinazolin-4-amine* (11626146) Yeild 57%. Mp. 115-8ºС (Hexane). Mass (EI), m/z (I*_relat_*.(%)): 247.3603 [M]^+^(70). C_13_H_17_N_3_S. ^1^H NMR (DMSO-d_6_ with 0.05% v/v TMS, 300 MHz,): δ 1.27 (6H, d, *J =*6.8, (CH_3_)_2_CH), 1.34 (3H, t, *J =* 7.3, CH_3_CH_2_S), 3.11 (2H, q, *J =* 7.3, CH_3_CH_2_S), 4.46 (H, oktet, *J =* 6.8, (CH_3_)_2_CH), 7.36 (1H, ddd, *J =* 1.2, 7.0, 8.2, H6), 7.49 (1H, dd, *J =* 1.2, 8.4, H8), 7.67 (1H, ddd, *J =* 1.4, 8.4, H7), 7.93 (1H, br d, *J =* 6.8, NH), 8.22 (1H, dd, *J =* 1.4, 8.2, H5) ppm.

*2-(ethylthio)-N-phenylquinazolin-4-amine* (11626147) Yeild 22%. Mp. 191-4ºС (H_2_O/*i*-PrOH). Mass (EI), m/z (I*_relat_*.(%)): 281.3765 [M]^+^(48). C_16_H_15_N_3_S. ^1^H NMR (DMSO-d_6_ with 0.05% v/v TMS, 300 MHz,): δ 1.32 (3H, t, *J =* 7.3, CH_3_CH_2_S), 3.10 (2H, q, *J =* 7.3, CH_3_CH_2_S), 7.16 (1H, t, *J =* 7.4, H4(Ph)), 7.46 (2H, t, *J =* 7.4, m-Ph), 7.50 (1H, t, *J =* 8.2, H6), 7.61 (1H, d, *J =* 8.2, H8), 7.75 – 7.88 (3H, m, H7, o-Ph), 8.48 (1H, d, *J =* 8.2, H5), 9.83 (1H, br s, NH) ppm.

*5-fluoro-N-methyl-2-(methylthio)quinazolin-4-amine* (11626164)Yeild 52%. Mp. 100-3ºС (Hexane). Mass (EI), m/z (I*_relat_*.(%)): 223.2710 [M]^+^(92). C_10_H_10_FN_3_S. ^1^H NMR (CDCl_3_,300 MHz,): δ 2.46 (3H, s, SCH_3_), 3.22 (3H, d, *J =* 4.4, NCH_3_), 6.88 (1H, br s, NH), 6.99 (1H, dd, *J =* 8.3, 12.3, H6), 7.52 – 7.63 (2H, m, H7, H8), ppm.

*N-ethyl-5-fluoro-2-(methylthio)quinazolin-4-amine* (11626165)Yeild 42%. Mp. 73-5ºС (Hexane). Mass (EI), m/z (I*_relat_*.(%)): 237.2976 [M]^+^(79). C_11_H_12_FN_3_S. ^1^H NMR (DMSO-d_6_ with 0.05% v/v TMS 300 MHz,): δ 1.23 (3H, t, *J =* 7.1, CH_3_CH_2_N), 2.52 (3H, s, SCH_3_), 3.54 (2H, dq, *J =* 5.4, 7.1, CH_3_CH_2_N), 7.15 (1H, ddd, *J =* 0.7, 8.2, 11.8, H6), 7.33 (1H, dd, *J =*0.7,8.2, H8), 7.66 (1H, dt, *J =* 6.3, 8.2, H7), 7.76 (1H, br s, NH) ppm.

*5-fluoro-N-isopropyl-2-(methylthio)quinazolin-4-amine* (11626167) Yeild 33%. Mp. 53-5ºС (H_2_O/EtOH). Mass (EI), m/z (I*_relat_*.(%)): 251.3242 [M]^+^(51). C_12_H_14_FN_3_S. ^1^H NMR (DMSO-d_6_ with 0.05% v/v TMS 300 MHz,): δ 1.27 (6H, d, *J =* 6.8, (CH_3_)_2_CH), 2.52 (3H, s, SCH_3_), 4.46 (H, oktet, *J =* 6.8, (CH_3_)_2_CH), 7.12 (1H, br s, NH), 7.18 (1H, dd, *J =* 8.2, 11.8, H6), 7.40 (1H, d, *J =* 8.2, H8), 7.66 (1H, q, *J =* 8.2, H7) ppm.

*5-fluoro-2-(methylthio)-N-phenylquinazolin-4-amine* (11626168) Yeild 16%. Mp. 82-5ºС (Hexane). Mass (EI), m/z (I*_relat_*.(%)): 285.3403 [M]^+^(56). C_15_H_12_FN_3_S. ^1^H NMR (DMSO-d_6_ with 0.05% v/v TMS, 300 MHz): δ 2.47 (3H, s, SCH_3_), 7.18 (1H, t, *J =* 7.3, p-Ph), 7.29 (1H, dd, *J =* 8.0, 12.3, H6), 7.40 (2H, t, *J =* 7.3, m-Ph), 7.45 (1H, d, *J =* 8.0, H8), 7.72 (2H, d, *J =* 7.3, o-Ph), 7.77 (1H, dt, *J =* 6.0, 8.4, H7), 9.14 (1H, br d, *J =* 13.2, NH) ppm.

*2-(ethylthio)-5-fluoro-N-methylquinazolin-4-amine* (11626244) Yeild 43%. Mp. 73-6ºС (Hexane). Mass (EI), m/z (I*_relat_*.(%)): 237.2976 [M]^+^ (37). C_11_H_12_FN_3_S. ^1^H NMR (DMSO-d_6_ with 0.05% v/v TMS, 300 MHz): δ 1.35 (3H, t, *J =* 7.3, CH_3_CH_2_), 3.0 (3H, d, *J =* 4.4, NCH_3_), 3.12 (2H, q, *J =* 7.3, CH_3_CH_2_), 7.15 (1H, ddd, *J =* 1.0, 8.0, 12.2, H6), 7.34 (1H, dd, *J =* 1.0, 8.4, H8), 7.66 (1H, dt, *J =* 6.2, 8.2, H7), 7.79 (1H, br dd , *J =* 4.4, 10.3 NH) ppm.

*5-fluoro-N,N-dimethyl-2-(methylthio)quinazolin-4-amine* (11626245) Yeild 26%. Mp. 70-3ºС (Hexane). Mass (EI), m/z (I*_relat_*.(%)): 237.2976 [M]^+^(43). C_11_H_12_FN_3_S. ^1^H NMR (DMSO-d_6_ with 0.05% v/v TMS, 300 MHz,): δ 2.53 (3H, s, SCH_3_), 3.12 (6H, d, *J =* 4.5, (CH_3_)_2_N), 7.15 (1H, ddd, *J =* 1.1, 8.0, 11.8, H6), 7.40 (1H, dd, *J =* 1.1, 8.4, H8), 7.71 (1H, dt, *J =* 6.0, 8.0, H7) ppm.

*5-fluoro-2-(methylthio)quinazolin-4-amine* (11626246) Yeild 52%. Mp. 190-2ºС (EtOH). Mass (EI), m/z (I*_relat_*.(%)): 209.2444 [M]^+^ (60). C_9_H_8_FN_3_S. ^1^H NMR (DMSO-d_6_ with 0.05% v/v TMS, 300 MHz,): δ 2.49 (3H, s, SCH_3_), 7.15 (1H, ddd, *J =* 1.0, 8.0, 11.9, H6), 7.36 (1H, dd, *J =* 1.0, 8.5, H8), 7.69 (1H, dt, *J =* 6.2, 8.2, H7), 8.01 (1H, br s, NH), ppm.

*2-(methylthio)quinazolin-4-amine* (11626247)Yeild 15%. Mp. 233-6ºС (Hexane/EtOAc). Mass (EI), m/z (I*_relat_*.(%)): 191.2540 [M]^+^ (36). C_9_H_9_N_3_S. ^1^H NMR (DMSO-d_6_ with 0.05% v/v TMS, 300 MHz,): δ 2.49 (3H, s, SCH_3_), 7.36 (1H, ddd, *J =* 1.3, 6.9, 8.2, H6), 7.53 (1H, dd, *J =* 1.2, 8.4, H8), 7.70 (1H, ddd, *J =* 1.4, 8.4, H7), 7.80 (2H, br s, NH_2_), 8.13 (1H, dd, *J =* 1.4, 8.2, H5) ppm.

*2-(ethylthio)-5-fluoroquinazolin-4-amine* (11626248)Yeild 58%. Mp. 158-160ºС (Hexane/EtOAc). Mass (EI), m/z (I*_relat_*.(%)): 223.2710 [M]^+^(47). C_10_H_10_FN_3_S. ^1^H NMR (DMSO-d_6_ with 0.05% v/v TMS, 300 MHz,): 1.33 (3H, t, *J =* 7.3, CH_3_CH_2_), 3.11 (2H, q, *J =* 7.3, CH_3_CH_2_), 7.15 (1H, ddd, *J =* 1.0, 8.0, 11.8, H6), 7.26 (1H, br s, NH), 7.35 (1H, dd, *J =* 1.0, 8.3, H8), 7.68 (1H, dt, *J =* 6.2, 8.2, H7), 8.01 (1H, br s, NH), ppm.

*2-(ethylthio)quinazolin-4-amine* (11626249)Yeild 56%. Mp. 147-150ºС (Cyclohexane). Mass (EI), m/z (I*_relat_*.(%)): 205.2806 [M]^+^ (58). C_10_H_11_N_3_S. ^1^H NMR (DMSO-d_6_ with 0.05% v/v TMS, 300 MHz,): δ 1.31 (3H, t, *J =* 7.3, CH_3_CH_2_), 3.10 (2H, q, *J =* 7.3, CH_3_CH_2_), 7.35 (1H, ddd, *J =* 1.3, 6.9, 8.2, H6), 7.50 (1H, dd, *J =* 1.2, 8.4, H8), 7.69 (1H, ddd, *J =* 1.4, 8.4, H7), 7.77 (2H, br s, NH_2_), 8.11 (1H, dd, *J =* 1.4, 8.2, H5) ppm.

*2-(benzylthio)-5-fluoro-N-methylquinazolin-4-amine* (11626250) Yeild 50%.Mp. 101-4ºС (Hexane). Mass (EI), m/z (I*_relat_*.(%)): 299.3670 [M]^+^ (61). C_16_H_14_FN_3_S. ^1^H NMR (DMSO-d_6_ with 0.05% v/v TMS, 300 MHz): δ 3.0 (3H, d, *J =* 4.4, NCH_3_), 4.44 (2H, s, PhCH_2_), 7.16 (1H, ddd, *J =* 1.0, 8.0, 12.2, H6), 7.22 (1H, t, p-Ph), 7.30 (2H, t, *J =* 7.3, m-Ph),7.39 (1H, dd, *J =* 1.0, 8.4, H8), 7.46 (2H, d, J=7.3, o-Ph), 7.68 (1H, dt, *J =* 6.2, 8.2, H7), 7.83 (1H, br dd , *J =* 4.4, 10.7 NH) ppm.

*2-(benzylthio)-N-ethyl-5-fluoroquinazolin-4-amine* (11626251)Yeild 43%. Mp. 79-82ºС (Hexane). Mass (EI), m/z (I*_relat_*.(%)): 313.3936 [M]^+^ (44). C_17_H_16_FN_3_S. ^1^H NMR (DMSO-d_6_ with 0.05% v/v TMS, 300 MHz): δ 1.18 (3H, t, *J =* 6.9, CH_3_CH_2_), 3.55 (2H, dq, *J =* 4.0, 6.9, CH_3_CH_2_), 4.43 (2H, s, PhCH_2_), 7.17 (1H, ddd, *J =* 1.0, 8.0, 12.3, H6), 7.21 - 7.34 (3H, m, m-Ph, p-Ph), 7.39 (1H, dd, *J =* 1.0, 8.4, H8), 7.43-7.48 (2H, m, o-Ph), 7.68 (1H, dt, *J =* 6.2, 8.2, H7), 7.79 (1H, br dt ,*J =* 11.3, 4.0 NH) ppm.

*2-(ethylthio)-8-fluoro-N-methylquinazolin-4-amine* (11626252)Yeild 72%.Mp. 204-7ºС (Hexane/EtOAc). Mass (EI), m/z (I*_relat_*.(%)): 237.2976 [M]^+^ (39). C_11_H_12_FN_3_S. ^1^H NMR (DMSO-d_6_, 300 MHz,): δ 1.35 (3H, t, *J =* 7.3, CH_3_CH_2_), 3.00 (3H, d, *J =* 4.5, NCH_3_), 3.14 (2H, q, *J =* 7.3, CH_3_CH_2_), 7.32 (1H, dt, *J =* 5.1, 8.1, H6), 7.51 (1H, dd, *J =* 7.8, 10.9, H7), 7.90 (1H, d, *J =* 8.3, H5), 8.41 (1H, br s, NH) ppm.

*N-ethyl-2-(ethylthio)-8-fluoroquinazolin-4-amine* (11626253) Yeild 47%. Mp. 136-9ºС (Hexane/EtOAc). Mass (EI), m/z (I*_relat_*.(%)): 251.3242 [M]^+^(59). C_12_H_14_FN_3_S. ^1^H NMR (DMSO-d_6_ with 0.05% v/v TMS, 300 MHz,): δ 1.23 (3H, t, *J =* 7.2, CH_3_CH_2_N), 1.35 (3H, t, *J =* 7.3, CH_3_CH_2_S), 3.13 (2H, q, *J =* 7.3, CH_3_CH_2_S), 3.55 (2H, dq, *J =* 5.3, 7.2, CH_3_CH_2_N), 7.33 (1H, dt, *J =* 5.1, 8.1, H6), 7.54 (1H, dd, *J =* 7.9, 10.9, H7), 7.96 (1H, d, *J =* 8.3, H5), 8.47 (1H, br t, *J =* 5.3, NH) ppm.

*2-(ethylthio)-7-fluoro-N-methylquinazolin-4-amine* (11626254) Yeild 27%. Mp. 134-7ºС (Hexane/EtOAc). Mass (EI), m/z (I*_relat_*.(%)): 237.2976 [M]^+^(52). C_11_H_12_FN_3_S. ^1^H NMR (DMSO-d_6_ with 0.05% v/v TMS, 300 MHz,): δ 1.34 (3H, t, *J =* 7.3, CH_3_CH_2_), 2.98 (3H, d, *J =* 4.5, NCH_3_), 3.12 (2H, q, *J =* 7.3, CH_3_CH_2_), 7.20 – 7.30 (2H, m, H6, H8), 8.18 (1H, dd, *J =* 6.1, 9.0, H5), 8.43 (1H, br m, NH) ppm.

*N-ethyl-2-(ethylthio)-7-fluoroquinazolin-4-amine* (11626255) Yeild 57%. Mp. 130-2ºС (Hexane/EtOAc). Mass (EI), m/z (I*_relat_*.(%)): 251.3242 [M]^+^ (57). C_12_H_14_FN_3_S. ^1^H NMR (DMSO-d_6_ with 0.05% v/v TMS, 300 MHz,): δ 1.23 (3H, t, *J =* 7.2, CH_3_CH_2_N), 1.33 (3H, t, *J =* 7.3, CH_3_CH_2_S), 3.11 (2H, q, *J =* 7.3, CH_3_CH_2_S), 3.53 (2H, dq, *J =* 5.4, 7.2, CH_3_CH_2_N), 7.21 – 7.30 (2H, m, H6, H8), 8.23 (1H, dd, *J =* 6.1, 9.0, H5), 8.40 (1H, br t, *J =* 5.4 NH) ppm.

*2-(ethylthio)-6-fluoro-N-methylquinazolin-4-amine* (11626256) Yeild 69%. Mp. 173-6ºС (Hexane/EtOAc). Mass (EI), m/z (I*_relat_*.(%)): 237.2976 [M]^+^(64). C_11_H_12_FN_3_S. ^1^H NMR (DMSO-d_6_, 300 MHz,): δ 1.33 (3H, t, *J =* 7.3, CH_3_CH_2_), 2.98 (3H, d, *J =* 4.5, NCH_3_), 3.11 (2H, q, *J =* 7.3, CH_3_CH_2_), 7.52 – 7.65 (2H, m, H7, H8), 7.92 (1H, dt, *J =* 9.9, 1.7, H5), 8.27 (1H, br m, NH) ppm.

*N-ethyl-2-(ethylthio)-6-fluoroquinazolin-4-amine* (11626257) Yeild 36%. Mp. 132-5ºС (Hexane). Mass (EI), m/z (I*_relat_*.(%)): 251.3242 [M]^+^(33). C_12_H_14_FN_3_S.^1^H NMR (DMSO-d_6_ with 0.05% v/v TMS, 300 MHz,): δ 1.23 (3H, t, *J =* 7.2, CH_3_CH_2_N), 1.34 (3H, t, *J =* 7.3, CH_3_CH_2_S), 3.11 (2H, q, *J =* 7.3, CH_3_CH_2_S), 3.53 (2H, dq, *J =* 5.5, 7.2, CH_3_CH_2_N), 7.50 – 7.65 (2H, m, H7, H8), 7.92 (1H, ddd, *J =* 1.1, 2.0, 9.9, H5), 8.27 (1H, br t, *J =* 5.5, NH) ppm.

*2-(ethylthio)-N-methyl-6-nitroquinazolin-4-amine* (11626258) Yeild 55%. Mp. 190-3ºС (Hexane/DCM). Mass (EI), m/z (I*_relat_*.(%)): 264.3047 [M]^+^(92). C_11_H_12_N_4_O_2_S. ^1^H NMR (DMSO-d_6_ with 0.05% v/v TMS, 300 MHz,): δ 1.36 (3H, t, *J =* 7.3, CH_3_CH_2_), 3.02 (3H, d, *J =* 4.5, NCH_3_), 3.16 (2H, q, *J =* 7.3, CH_3_CH_2_), 7.61 (1H, d, *J =* 9.2, H8), 8.40 (1H, dd, *J =* 2.4, 9.2, H7), 9.03 (1H, br m, NH), 9.19 (1H, d, J =2.4 , H5) ppm.

*N-ethyl-2-(ethylthio)-6-nitroquinazolin-4-amine* (11626259) Yeild 39%. Mp. 161-4ºС (Hexane/DCM). Mass (EI), m/z (I*_relat_*.(%)): 278.3313 [M]^+^(79). C_12_H_14_N_4_O_2_S. ^1^H NMR (DMSO-d_6_, 300 MHz,):δ 1.25 (3H, t, *J =* 7.2, CH_3_CH_2_N), 1.35 (3H, t, *J =* 7.3, CH_3_CH_2_S), 3.14 (2H, q, *J =* 7.3, CH_3_CH_2_S), 3.56 (2H, dq, *J =* 5.5, 7.2, CH_3_CH_2_N), 7.59 (1H, d, *J =* 9.2, H8), 8.39 (1H, dd, *J =* 2.4, 9.2, H7), 9.01 (1H, br t, *J =* 5.5, NH), 9.25 (1H, d, J =2.4 , H5) ppm.

*2-(ethylthio)-N-methyl-6-(trifluoromethyl)quinazolin-4-amine* (11726001) Yeild 50%. Mp. 161-3ºС (Hexane). Mass (EI), m/z (I*_relat_*.(%)): 287.3051 [M]^+^(56). C_12_H_12_F_3_N_3_S. ^1^H NMR (DMSO-d_6_ with 0.05% v/v TMS, 300 MHz,): δ 1.35 (3H, t, *J =* 7.3, CH_3_CH_2_), 3.00 (3H, d, *J =* 4.4, NCH_3_), 3.14 (2H, q, *J =* 7.3, CH_3_CH_2_), 7.65 (1H, d, *J =* 8.7, H8), 7.94 (1H, d, *J =* 8.7, H7) , 8.61 (1H, s, H5), 9.03 (1H, br m, NH) ppm.

*N-ethyl-2-(ethylthio)-6-(trifluoromethyl)quinazolin-4-amine* (11726002) Yeild 56%. Mp. 162-5ºС (Hexane). Mass (EI), m/z (I*_relat_*.(%)): 301.3317 [M]^+^(91). C_13_H_14_F_3_N_3_S. ^1^H NMR (DMSO-d_6_ with 0.05% v/v TMS, 300 MHz,): δ 1.25 (3H, t, *J =* 7.2, CH_3_CH_2_N), 1.35 (3H, t, *J =* 7.3, CH_3_CH_2_S), 3.13 (2H, q, *J =* 7.3, CH_3_CH_2_S), 3.57 (2H, dq, *J =* 5.3, 7.2, CH_3_CH_2_N), 7.64 (1H, d, *J =* 8.8, H8), 7.93 (1H, d, *J =* 8.8, H7), 8.67 (1H, s, H5), 8.72 (1H, br m, NH) ppm.

*N-methyl-2-(propylthio)quinazolin-4-amine* (11726003) Yeild 49%. Mp. 124-7ºС (Hexane/EtOAc). Mass (EI), m/z (I*_relat_*.(%)): 233.3337 [M]^+^(31). C_12_H_15_N_3_S. ^1^H NMR (DMSO-d_6_ with 0.05% v/v TMS, 300 MHz,): δ 1.01 (3H, t, *J =* 7.3, CH_3_CH_2_), 1.72 (3H, hex, *J =* 7.3, CH_3_CH_2_), 2.99 (3H, d, *J =* 4.5, NCH_3_), 3.12 (2H, t, *J =* 7.3, CH_2_CH_2_S), 7.36 (1H, dd, 7.0, 8.3, H6), 7.50 (1H, d, *J =* 8.4, H8), 7.67 (1H, dd, *J =* 7.0, 8.4, H8), 8.09 (1H, d, *J =* 8.3, H5), 8.31 (1H, br m, NH) ppm.

*N-ethyl-2-(propylthio)quinazolin-4-amine* (11726004) Yeild 58%. Mp. 128-130ºС (Hexane/EtOAc). Mass (EI), m/z (I*_relat_*.(%)): 247.3603 [M]^+^(45). C_13_H_17_N_3_S. . H NMR (DMSO-d_6_ with 0.05% v/v TMS, 300 MHz,): δ 1.00 (3H, t, *J =* 7.3, CH_3_CH_2_), 1.23 (3H, t, *J =* 7.2, CH_3_CH_2_N), 1.72 (2H, hex, *J =* 7.3, CH_3_CH_2_), 3.10 (2H, t, *J =* 7.3, CH_2_CH_2_S), 3.54 (2H, dq, *J =* 5.3, 7.2, CH_3_CH_2_N), 7.36 (1H, dd, 7.0, 8.3, H6), 7.49 (1H, d, *J =* 8.4, H8), 7.67 (1H, dd, *J =* 7.0, 8.4, H8), 8.12 (1H, d, *J =* 8.3, H5), 8.26 (1H, br m, NH) ppm.

*2-(ethylthio)-N-methyl-8-(trifluoromethyl)quinazolin-4-amine hydrochloride* (11726027) Yeild 65%. Mp. 127-130ºС. Mass (EI), m/z (I*_relat_*.(%)): 323.7657 [M]^+^(97). C_12_H_13_ClF_3_N_3_S. ^1^H NMR (DMSO-d_6_with 0.05% v/v TMS, 300 MHz,): δ 1.35 (3H, t, *J =* 7.2, CH_3_CH_2_), 2.99 (3H, d, *J =* 3.1, NCH_3_), 3.11 (2H, q, *J =* 7.2, CH_3_CH_2_), 7.48 (1H, dd, *J =* 7.5, 8.2, H6), 8.06 (1H, d, *J =* 7.5, H7) , 8.43 (1H, d, *J =* 8.2, H5), 8.70 (1H, br s, NH) ppm.

*N-ethyl-2-(ethylthio)-8-(trifluoromethyl)quinazolin-4-amine* (11726028) Yeild 66%. Mp. 71-4ºС (Hexane). Mass (EI), m/z (I*_relat_*.(%)): 301.3317 [M]^+^(48). C_13_H_14_F_3_N_3_S. ^1^H NMR (DMSO-d_6_ with 0.05% v/v TMS, 300 MHz,): δ 1.23 (3H, t, *J =* 7.2, CH_3_CH_2_N), 1.35 (3H, t, *J =* 7.2, CH_3_CH_2_S), 3.10 (2H, q, *J =* 7.2, CH_3_CH_2_S), 3.54 (2H, dq, *J =* 5.3, 7.2, CH_3_CH_2_N), 7.48 (1H, dd, *J =* 7.5, 8.1, H6), 8.05 (1H, d, *J =* 7.5, H7) , 8.42 (1H, d, *J =* 8.1, H5), 8.52 (1H, br s, NH) ppm.

*8-fluoro-N-methyl-2-(propylthio)quinazolin-4-amine* (11726029) Yeild 64%. Mp. 153-6ºС (Hexane/EtOAc). Mass (EI), m/z (I*_relat_*.(%)): 251.3242 [M]^+^ (31). C_12_H_14_FN_3_S. ^1^H NMR (DMSO-d_6_ with 0.05% v/v TMS, 300 MHz,): δ 1.00 (3H, t, *J =* 7.3, CH_3_CH_2_), 1.73 (2H, hex, *J =* 7.3, CH_3_CH_2_), 3.00 (3H, d, *J =* 4.4, NCH_3_), 3.13 (2H, t, *J =* 7.3, CH_2_CH_2_S), 7.33 (1H, dt, *J =* 5.0, 8.0, H6), 7.52 (1H, dd, *J =* 8.0, 10.8, H7), 7.90 (1H, d, *J =* 8.3, H5), 8.43 (1H, br s, NH) ppm.

*N-ethyl-8-fluoro-2-(propylthio)quinazolin-4-amine* (11726030) Yeild 57%. Mp. 160-3ºС (Hexane/EtOAc). Mass (EI), m/z (I*_relat_*.(%)): 265.3508 [M]^+^(40). C_13_H_16_FN_3_S. ^1^H NMR (DMSO-d_6_ with 0.05% v/v TMS, 300 MHz,): δ 1.00 (3H, t, *J =* 7.3, CH_3_CH_2_), 1.23 (3H, t, *J =* 7.2, CH_3_CH_2_N), 1.73 (2H, hex, *J =* 7.3, CH_3_CH_2_), 3.12 (2H, t, *J =* 7.3, CH_2_CH_2_S), 3.55 (2H, dq, *J =* 5.3, 7.2, CH_3_CH_2_N), 7.32 (1H, dt, *J =* 5.1, 8.1, H6), 7.52 (1H, dd, *J =* 7.8, 10.9, H7), 7.95 (1H, d, *J =* 8.3, H5), 8.41 (1H, br s, NH) ppm.

*2-(isopropylthio)-N-methylquinazolin-4-amine* (11726037) Yeild 54%. Mp. 134-6ºС (Hexane/EtOAc). Mass (EI), m/z (I*_relat_*.(%)): 233.3337 [M]^+^(86). C_12_H_15_N_3_S. ^1^H NMR (DMSO-d_6_ with 0.05% v/v TMS, 300 MHz,): δ 1.39 (6H, d, *J =* 6.9, SCH(CH_3_)_2_, 2.99 (3H, d, *J =* 4.4, NCH_3_), 3.96 (1H, hept, *J =* 6.9, SCH), 7.37 (1H, ddd, *J =* 1.2, 7.0, 8.2, H6), 7.50 (1H, dd, *J =* 1.2, 8.3, H8), 7.68 (1H, ddd, *J =* 1.4, 7.0, 8.3, H7), 8.08 (1H, dd, *J =* 1.4, 8.2, H5), 8.32 (1H, br s, NH) ppm.

*N-ethyl-2-(isopropylthio)quinazolin-4-amine* (11726038) Yeild 42%. Mp. 129-132ºС (Hexane/EtOAc). Mass (EI), m/z (I*_relat_*.(%)): 247.3603 [M]^+^(48). C_13_H_17_N_3_S. ^1^H NMR (DMSO-d_6_ with 0.05% v/v TMS, 300 MHz,): δ 1.23 (3H, t, *J =* 7.2, CH_3_CH_2_N), 1.39 (6H, d, *J =* 6.8, SCH(CH_3_)_2_), 3.53 (2H, dq, *J =* 5.3, 7.2, CH_3_CH_2_N), 3.93 (1H, hept, *J =* 6.8, SCH), 7.36 (1H, ddd, *J =* 1.2, 7.0, 8.2, H6), 7.49 (1H, dd, *J =* 1.2, 8.3, H8), 7.67 (1H, ddd, *J =* 1.4, 7.0, 8.3, H7), 8.14 (1H, dd, *J =* 1.4, 8.2, H5), 8.31 (1H, br s, NH) ppm.

*8-fluoro-2-(isopropylthio)-N-methylquinazolin-4-amine* (11726039) Yeild 65%. Mp. 179-181ºС (Hexane/EtOAc). Mass (EI), m/z (I*_relat_*.(%)): 251.3242 [M]^+^(48). C_12_H_14_FN_3_S. ^1^H NMR (DMSO-d_6_ with 0.05% v/v TMS, 300 MHz,): δ 1.41 (6H, d, *J =* 6.8, SCH(CH_3_)_2_), 3.00 (3H, d, *J =* 4.5, NCH_3_), 3.95 (1H, hept, *J =* 6.8, SCH), 7.33 (1H, dt, *J =* 5.0, 8.2, H6), 7.52 (1H, dd, *J =* 7.8, 10.8, H7), 7.90 (1H, d, *J =* 8.2, H5), 8.42 (1H, br s, NH) ppm.

*N-ethyl-8-fluoro-2-(isopropylthio)quinazolin-4-amine* (11726040)Yeild 74%. Mp. 165-8ºС (Hexane/EtOAc). Mass (EI), m/z (I*_relat_*.(%)): 265.3508 [M]^+^(49). C_13_H_16_FN_3_S. ^1^H NMR (DMSO-d_6_ with 0.05% v/v TMS, 300 MHz,): δ 1.23 (3H, t, *J =* 7.2, CH_3_CH_2_N), 1.40 (6H, d, *J =* 6.8, SCH(CH_3_)_2_), 3.54 (2H, dq, *J =* 5.3, 7.2, CH_3_CH_2_N), 3.94 (1H, hept, *J =* 6.8, SCH), 7.33 (1H, dt, *J =* 5.0, 8.2, H6), 7.53 (1H, dd, *J =* 7.9, 10.8, H7), 7.95 (1H, d, *J =* 8.3, H5), 8.43 (1H, br s, NH) ppm.

*2-(ethylthio)-N^4^-methylquinazoline-4,6-diamine hydrochloride* (11726077) Yeild 26%. Mp. 200-3ºС (MeOH). Mass (EI), m/z (I*_relat_*.(%)): 270.7825 [M]^+^(39). C_11_H_15_ClN_4_S. ^1^H NMR (DMSO-d_6_ with 0.05% v/v TMS, 300 MHz,): δ 1.41 (3H, t, *J =* 7.3, CH_3_CH_2_), 3.16 (3H, d, *J =* 4.4, NCH_3_), 3.34 (2H, q, *J =* 7.3, CH_3_CH_2_), 7.42 (1H, dd, *J =* 2.8, 8.8, H7), 7.54 (1H, d, *J =* 2.8, H5), 7.61 (1H, d, *J =* 8.8, H8), 10.04 (1H, br s, NH) ppm.

*N-[2-(ethylthio)-4-(methylamino)quinazolin-6-yl]acetamide* (11726078) Yeild 49%. Mp. 109-112ºС. Mass (EI), m/z (I*_relat_*.(%)): 276.3585 [M]^+^(92). C_11_H_16_N_4_OS. ^1^H NMR (DMSO-d_6_ with 0.05% v/v TMS, 300 MHz,): δ 1.41 (3H, t, *J =* 7.3, CH_3_CH_2_), 2.10 (3H, s, CH_3_CO), 2.98 (3H, d, *J =* 4.4, NCH_3_), 3.25 (2H, q, *J =* 7.3, CH_3_CH_2_), 7.42 (1H, d, *J =* 8.8, H8), 7.62 (1H, d, *J =* 8.8, H7), 8.18 (1H, br s, NHCH_3_), 8.30 (1H, s, H5), 10.08 (1H, br s, NH) ppm.

*2-(ethylthio)-N-methyl-6,8-dinitroquinazolin-4-amine* (11726080) Yeild 33%. Mp. 252-5ºС (Hexane/EtOAc). Mass (EI), m/z (I*_relat_*.(%)): 309.3023 [M]^+^(43). C_11_H_11_N_5_O_4_S. ^1^H NMR (DMSO-d_6_ with 0.05% v/v TMS, 300 MHz,): δ 1.33 (3H, t, *J =* 7.2, CH_3_CH_2_), 3.03 (3H, d, *J =* 4.2, NCH_3_), 3.08 (2H, q, *J =* 7.2, CH_3_CH_2_), 8.99 (1H, d, J =2.3, H5), 9.38 (1H, d, *J =* 2.3, H7), 9.40 (1H, br s, NH) ppm.

*2-(ethylthio)-6,7-difluoro-N-methylquinazolin-4-amine* (11726142)Yeild 72%. Mp. 163-5ºС (Hexane/EtOAc). Mass (EI), m/z (I*_relat_*.(%)): 255.2881 [M]^+^(63). C_11_H_11_F_2_N_3_S. ^1^H NMR (DMSO-d_6_ with 0.05% v/v TMS, 300 MHz,): δ 1.34 (3H, t, *J =* 7.3, CH_3_CH_2_), 2.98 (3H, d, *J =* 4.4, NCH_3_), 3.11 (2H, q, *J =* 7.3, CH_3_CH_2_), 7.50 (1H, dd, J =7.8, 11.9, H5), 8.17 (1H, dd, *J =* 8.6, 11.6, H8), 8.35 (1H, br s, NH) ppm.

*2-(ethylthio)-6,7,8-trifluoro-N-methylquinazolin-4-amine* (11726146) Yeild 68%. Mp. 160-2ºС (Petroleum ether (70/100)/acetone).Mass (EI), m/z (I*_relat_*.(%)): 273.2785 [M]^+^(37). C_11_H_10_F_3_N_3_S. ^1^H NMR (DMSO-d_6_ with 0.05% v/v TMS, 300 MHz,): δ 1.35 (3H, t, *J =* 7.3, CH_3_CH_2_), 2.98 (3H, d, *J =* 4.5, NCH_3_), 3.13 (2H, q, *J =* 7.3, CH_3_CH_2_), 8.03 (1H, ddd, *J =* 2.3, 7.9, 11.5, H8), 8.46 (1H, br s, NH) ppm.

*8-chloro-2-(ethylthio)-N-methylquinazolin-4-amine* (11726147) Yeild 58%. Mp. 164-7ºС (Hexane/EtOAc). Mass (EI), m/z (I*_relat_*.(%)): 253.7519 [M]^+^(47). C_11_H_12_ClN_3_S. ^1^H NMR (DMSO-d_6_ with 0.05% v/v TMS, 300 MHz,): δ 1.38 (3H, t, *J =* 7.3, CH_3_CH_2_), 3.00 (3H, d, *J =* 3.1, NCH_3_), 3.16 (2H, q, *J =* 7.3, CH_3_CH_2_), 7.33 (1H, dd, *J =* 7.7, 8.3, H6), 8.06 (1H, dd, *J =* 1.2, 7.7, H7) , 8.43 (1H, dd, *J =* 1.2, 8.3, H5), 8.48 (1H, br s, NH) ppm.

*2-(ethylthio)-7,8-difluoro-N-methylquinazolin-4-amine* (11726148) Yeild 64%. Mp. 186-9ºС (Hexane/EtOAc). Mass (EI), m/z (I*_relat_*.(%)): 255.2881 [M]^+^(56). C_11_H_11_F_2_N_3_S. ^1^H NMR (DMSO-d_6_ with 0.05% v/v TMS, 300 MHz,): δ 1.36 (3H, t, *J =* 7.3, CH_3_CH_2_), 3.00 (3H, d, *J =* 4.5, NCH_3_), 3.15 (2H, q, *J =* 7.3, CH_3_CH_2_), 7.42 (1H, dt, J =7.4, 9.6, H6), 8.96 (1H, ddd, *J =* 2.1, 5.3, 9.6, H5), 8.53 (1H, br s, NH) ppm.

*6-bromo-2-(ethylthio)-N-methylquinazolin-4-amine* (11826051) Yeild 46%. Mp. 173-6ºС (Petroleum ether (70/100)/acetone).Mass (EI), m/z (I*_relat_*.(%)): 298.2032 [M]^+^(39). C_11_H_12_BrN_3_S. ^1^H NMR (DMSO-d_6_ with 0.05% v/v TMS, 300 MHz,): δ 1.34 (3H, t, *J =* 7.3, CH_3_CH_2_), 2.99 (3H, d, *J =* 4.4, NCH_3_), 3.14 (2H, q, *J =* 7.3, CH_3_CH_2_), 7.44 (1H, d, *J =* 8.3, H8), 7.79 (1H, d, *J =* 8.3, H7) , 8.37 (1H, s, H5), 8.42 (1H, br m, NH) ppm.

*2-(ethylthio)-N,6-dimethylquinazolin-4-amine* (11826052) Yeild 64%. Mp. 165-8ºС (Hexane/EtOAc). Mass (EI), m/z (I*_relat_*.(%)): 298.2032 [M]^+^(43). C_12_H_15_N_3_S. ^1^H NMR (DMSO-d_6_ with 0.05% v/v TMS, 300 MHz,): δ 1.34 (3H, t, *J =* 7.3, CH_3_CH_2_), 2.42 (3H, s, C6CH_3_), 2.99 (3H, d, *J =* 4.4, NCH_3_), 3.14 (2H, q, *J =* 7.3, CH_3_CH_2_), 7.41 (1H, d, *J =* 9.0, H8), 7.52 (1H, d, *J =* 9.0, H7), 7.88 (1H, s, H5), 8.17 (1H, br m, NH) ppm.

*2-(ethylthio)-N-methyl-6-phenylquinazolin-4-amine*(11826053)Yeild 44%.Mp. 226-8ºС (EtOH). Mass (EI), m/z (I*_relat_*.(%)): 295.4031 [M]^+^(97). C_17_H_17_N_3_S. ^1^H NMR (DMSO-d_6_ with 0.05% v/v TMS, 300 MHz,): δ 1.34 (3H, t, *J =* 7.3, CH_3_CH_2_), 2.99 (3H, d, *J =* 4.4, NCH_3_), 3.14 (2H, q, *J =* 7.3, CH_3_CH_2_), 7.39 (1H, t, J =7.3, p-Ph), 7.51 (2H, t, J = 7.3, m-Ph), 7.58 (1H, d, *J =* 8.8, H8), 7.80 (2H, t, J = 7.3, o-Ph), 8.02 (1H, dd, *J =* 2.0, 8.8, H7), 8.43 (1H, d, J = 2.0, H5), 8.46 (1H, br m, NH) ppm.

*2-(ethylthio)-6-(4-fluorophenyl)-N-methylquinazolin-4-amine* (11826054) Yeild 52%. Mp. 228-231ºС (EtOH). Mass (EI), m/z (I*_relat_*.(%)): 313.3936 [M]^+^ (97). C_17_H_16_FN_3_S. ^1^H NMR (DMSO-d_6_ with 0.05% v/v TMS, 300 MHz,): δ 1.35 (3H, t, *J =* 7.3, CH_3_CH_2_), 3.03 (3H, d, *J =* 4.4, NCH_3_), 3.16 (2H, q, *J =* 7.3, CH_3_CH_2_), 7.30 (2H, t, *J =* 7.3, ArFH2), 7.65 (1H, d, *J =* 8.7, H8), 7.80 (2H dd, *J =* 6.5, 7.3, ArFH3), 7.96 (1H, d, *J =* 8.7, H7) , 8.45 (1H, s, H5), 8.47 (1H, br m, NH) ppm.

*6-(3,4-difluorophenyl)-2-(ethylthio)-N-methylquinazolin-4-amine* (11826055) Yeild 52%. Mp. 228-230ºС (EtOH). Mass (EI), m/z (I*_relat_*.(%)): 331.3840 [M]^+^(67). C_17_H_15_F_2_N_3_S. ^1^H NMR (DMSO-d_6_ with 0.05% v/v TMS, 300 MHz,): δ 1.35 (3H, t, *J =* 7.3, CH_3_CH_2_), 3.03 (3H, d, *J =* 4.4, NCH_3_), 3.16 (2H, q, *J =* 7.3, CH_3_CH_2_), 7.50 – 7.70 (3H, m, H8, 2ArF2), 7.87 (1H, ddd, *J =* 2.2, 7.7, 9.8, ArF2), 8.05(1H, dd, *J =* 2.2, 8.7, H7) , 8.45 (1H, s, H5), 8.48 (1H, br m, NH) ppm.

*2-(ethylthio)-N-methyl-6-[4-(trifluoromethyl)phenyl]quinazolin-4-amine* (11826056) Yeild 45%. Mp. 243-5ºС (EtOH). Mass (EI), m/z (I*_relat_*.(%)): 363.4011 [M]^+^(64). C_18_H_16_F_3_N_3_S. ^1^H NMR (DMSO-d_6_ with 0.05% v/v TMS, 300 MHz,): δ 1.35 (3H, t, *J =* 7.3, CH_3_CH_2_), 3.04 (3H, d, *J =* 4.4, NCH_3_), 3.16 (2H, q, *J =* 7.3, CH_3_CH_2_), 7.61 (1H, d, *J =* 8.7, H8), 7.86 (2H, d, J =8.2, ArCF3), 8.02 (2H, d, *J =* 8.2, ArCF3), 8.90 (1H, dd, *J =* 2.0, 8.7, H7), 8.50 (1H, br m, NH), 8.55 (1H, s, H5) ppm.

*6-(2,4-difluorophenyl)-2-(ethylthio)-N-methylquinazolin-4-amine* (11826057) Yeild 42%. Mp. 184-6ºС (EtOH). Mass (EI), m/z (I*_relat_*.(%)):331.3840 [M]^+^(39). C_17_H_15_F_2_N_3_S. ^1^H NMR (DMSO-d_6_ with 0.05% v/v TMS, 300 MHz,): δ 1.35 (3H, t, *J =* 7.3, CH_3_CH_2_), 3.04 (3H, d, *J =* 4.4, NCH_3_), 3.16 (2H, q, *J =* 7.3, CH_3_CH_2_), 7.23 (1H, t, J = 10, FPhH4), 7.36(1H, t, J = 10, FPhH5), 7.58 (1H, d, *J =* 8.7, H8), 7.66 (1H, dt, J = 6.5, 9.0, FPhH2), 7.51 (1H, td, J = 2.0, 8.9, H7), 8.30 (1H, s, H5), 8.46 (1H, br m, NH) ppm.

*2-(ethylthio)-N-methyl-7-(trifluoromethyl)quinazolin-4-amine* (11826058) Yeild 54%. Mp. 167-9ºС (Petroleum ether (70/100)/acetone). Mass (EI), m/z (I*_relat_*.(%)): 287.3051 [M]^+^(87). C_12_H_12_F_3_N_3_S. ^1^H NMR (DMSO-d_6_, 300 MHz,):δ 1.35 (3H, t, *J =* 7.2, CH_3_CH_2_), 2.99 (3H, d, *J =* 3.1, NCH_3_), 3.11 (2H, q, *J =* 7.2, CH_3_CH_2_), 7.65 (1H, d, *J =* 9.4, H6), 7.78 (1H, s, H8), 8.32 (1H, d, *J =* 9.4, H5), 8.59 (1H, br s, NH) ppm.

*2-(ethylthio)-N,8-dimethylquinazolin-4-amine* (11826059) Yeild 27%. Mp. 92-5ºС (Petroleum ether 70/100). Mass (EI), m/z (I*_relat_*.(%)): 233.3337 [M]^+^(57). C_12_H_15_N_3_S. ^1^H NMR (DMSO-d_6_, 300 MHz,):δ 1.37 (3H, t, *J =* 7.3, CH_3_CH_2_), 2.50 (3H, s, C8CH3), 2.97 (3H, d, *J =* 4.5, NCH_3_), 3.14 (2H, q, *J =* 7.3, CH_3_CH_2_), 7.24 (1H, dd, *J =* 7.1, 8.2, H6), 7.53 (1H, d, *J =* 7.1, H7), 7.90 (1H, d, *J =* 8.2, H5), 8.16 (1H, br s, NH) ppm.

*8-ethoxy-2-(ethylthio)-7-fluoro-N-methylquinazolin-4-amine* (11826060) Yeild 71%. Mp. 139-142ºС (Hexane/EtOAc). Mass (EI), m/z (I*_relat_*.(%)): 281.3502 [M]^+^(36). C_13_H_16_FN_3_OS. ^1^H NMR (DMSO-d_6_, 300 MHz,):δ 1.31 (3H, t, *J =* 7.3, CH_3_CH_2_S), 1.36 (3H, t, *J =* 7.2, CH_3_CH_2_O), 2.96 (3H, d, *J =* 4.5, NCH_3_), 3.13 (2H, q, *J =* 7.3, CH_3_CH_2_S), 4.31 (2H, q, *J =* 7.2, CH_3_CH_2_O), 7.28 (1H, t, *J =* 9.4, H6), 7.85 (1H, dd, *J =* 5.4, 9.4, H5), 8.32 (1H, br s, NH) ppm.

*7-bromo-2-(ethylthio)-N-methylquinazolin-4-amine* (11826061) Yeild 63%. Mp. 195-8ºС (Hexane/EtOAc ). Mass (EI), m/z (I*_relat_*.(%)): 298.2032 [M]^+^(61). C_11_H_12_BrN_3_S. ^1^H NMR (DMSO-d_6_, 300 MHz,): δ 1.35 (3H, t, *J =* 7.2, CH_3_CH_2_), 2.98 (3H, d, *J =* 4.4, NCH_3_), 3.16 (2H, q, *J =* 7.2, CH_3_CH_2_), 7.51 (1H, d, *J =* 8.8, H6), 7.67 (1H, s, H8), 8.03 (1H, d, *J =* 8.8, H5), 8.42 (1H, br s, NH) ppm.

*2-(ethylthio)-4-(methylamino)quinazoline-7-carbonitrile* (11826062) Yeild 61%. Mp. 192-5ºС (Hexane/EtOAc). Mass (EI), m/z (I*_relat_*.(%)): 244.3166 [M]^+^(37). C_12_H_12_N_4_S. ^1^H NMR (DMSO-d_6_, 300 MHz,):δ 1.34 (3H, t, *J =* 7.3, CH_3_CH_2_), 3.00 (3H, d, *J =* 4.6, NCH_3_), 3.16 (2H, q, *J =* 7.3, CH_3_CH_2_), 7.69 (1H, d, *J =* 8.4, H6), 7.95 (1H, s, H8), 8.23 (1H, d, *J =* 8.4, H5), 8.62 (1H, br s, NH) ppm.

*7-chloro-2-(ethylthio)-8-methoxy-N-methylquinazolin-4-amine* (11826063) Yeild 68%. Mp. 135-8ºС (Hexane/EtOAc). Mass (EI), m/z (I*_relat_*.(%)): 283.7779 [M]^+^(48). C_12_H_14_ClN_3_OS. ^1^H NMR (DMSO-d_6_, 300 MHz,): δ 1.36 (3H, t, *J =* 7.3, CH_3_CH_2_), 2.97 (3H, d, *J =* 4.6, NCH_3_), 3.22 (2H, q, *J =* 7.3, CH_3_CH_2_), 4.03 (3H, s, CH_3_O), 7.39 (1H, d, *J =* 8.9, H6), 7.82 (1H, d, *J =* 8.9, H5), 8.34 (1H, br s, NH) ppm.

*7-chloro-2-(ethylthio)-N-methylquinazolin-4-amine* (11826064) Yeild 63%. Mp. 179-182ºС (Hexane/EtOAc). Mass (EI), m/z (I*_relat_*.(%)): 253.7519 [M]^+^(43). C_11_H_12_ClN_3_S. ^1^H NMR (DMSO-d_6_, 300 MHz,): δ 1.34 (3H, t, *J =* 7.3, CH_3_CH_2_), 2.98 (3H, d, *J =* 4.5, NCH_3_), 3.11 (2H, q, *J =* 7.3, CH_3_CH_2_), 7.39 (1H, d, *J =* 8.8, H6), 7.51 (1H, s, H8), 8.10 (1H, d, *J =* 8.8, H5), 8.42 (1H, br s, NH) ppm.

*2-(ethylthio)-8-fluoro-N,7-dimethylquinazolin-4-amine* (11826065) Yeild 56%. Mp. 201-4ºС (Petroleum ether (70/100)/acetone). Mass (EI), m/z (I*_relat_*.(%)): 251.3242 [M]^+^(37). C_12_H_14_FN_3_S. ^1^H NMR (DMSO-d_6_, 300 MHz,): δ 1.35 (3H, t, *J =* 7.3, CH_3_CH_2_), 2.35 (3H, d, *J =* 2.2, C7CH3), 2.98 (3H, d, *J =* 4.4, NCH_3_), 3.13 (2H, q, *J =* 7.3, CH_3_CH_2_), 7.22 (1H, dd *J =* 7.2, 8.4, H6), 7.79 (1H, d, *J =* 8.4, H5), 8.31 (1H, br s, NH) ppm.

*8-chloro-2-(ethylthio)-6,7-difluoro-N-methylquinazolin-4-amine* (11826066) Yeild 70%. Mp. 75-8ºС (Petroleum ether (70/100)/acetone). Mass (EI), m/z (I*_relat_*.(%)): 289.7328 [M]^+^(47). C_11_H_10_ClF_2_N_3_S. ^1^H NMR (DMSO-d_6_, 300 MHz,): δ 1.36 (3H, t, *J =* 7.3, CH_3_CH_2_), 2.97 (3H, d, *J =* 4.5, NCH_3_), 3.13 (2H, q, *J =* 7.3, CH_3_CH_2_), 8.13 (1H, dd, *J =* 8.4, 11.3, H5), 8.46 (1H, br s, NH) ppm.

*2-(ethylthio)-N-methyl-7-phenylquinazolin-4-amine*(11826068)Yeild 40%.Mp. 188-191ºС (EtOH). Mass (EI), m/z (I*_relat_*.(%)): 295.4031 [M]^+^(53). C_17_H_17_N_3_S. ^1^H NMR (DMSO-d_6_, 300 MHz,): δ 1.36 (3H, t, *J =* 7.3, CH_3_CH_2_), 3.00 (3H, d, *J =* 4.3, NCH_3_), 3.15 (2H, q, *J =* 7.3, CH_3_CH_2_), 7.43 (1H , t, *J =* 7.3, p-Ph), 7.51 (2H, t, *J =* 7.3, m-Ph), 7.71 (1H, dd, *J =* 2.0, 8.5, H6), 7.75 (1H, d, *J =* 2.0, H8), 7.81 (2H, d, J=7.3, o-Ph), 8.18 (1H, d, *J =* 8.5, H5), 8.38 (1H, br s, NH) ppm.

*2-(ethylthio)-7-(4-fluorophenyl)-N-methylquinazolin-4-amine*(11826069)Yeild 36%. Mp. 186-9ºС (EtOH).Mass (EI), m/z (I*_relat_*.(%)): 313.3936 [M]^+^ (39). C_17_H_16_FN_3_S. ^1^H NMR (DMSO-d_6_, 300 MHz,): δ 1.36 (3H, t, *J =* 7.3, CH_3_CH_2_), 3.00 (3H, d, *J =* 4.3, NCH_3_), 3.14 (2H, q, *J =* 7.3, CH_3_CH_2_), 7.32 (2H , t, *J =* 8.5, FPh), 7.68 (1H, d, *J =* 8.7, H6), 7.73 (1H, s, H8), 7.86 (2H, dd, J=5.4, 8.5, FPh), 8.17 (1H, d, *J =* 8.3, H5), 8.38 (1H, br s, NH) ppm.

*7-(benzyloxy)-2-(ethylthio)-N-methylquinazolin-4-amine*(11826070) Yeild 24%. Mp. 170-3ºС (Hexane/EtOAc). Mass (EI), m/z (I*_relat_*.(%)): 325.4291 [M]^+^(37). C_18_H_19_N_3_OS. ^1^H NMR (DMSO-d_6_, 300 MHz,): δ 1.34 (3H, t, *J =* 7.3, CH_3_CH_2_), 2.96 (3H, d, *J =* 4.4, NCH_3_), 3.11 (2H, q, *J =* 7.3, CH_3_CH_2_), 5.22 (2H, s, CH_2_O), 6.97 – 7.09 (2H , m, H6, H8), 7.34 (1H, t, *J =* 7.3, p-Ph), 7.41 (2H, t, *J =* 7.3, m-Ph), 7.48 (2H, d, J=7.3, o-Ph), 8.00 (1H, d, *J =* 8.8, H5), 8.10 (1H, br s, NH) ppm.

*7-(3,4-difluorophenyl)-2-(ethylthio)-N-methylquinazolin-4-amine*(11826071)Yeild 26%. Mp. 201-4ºС (EtOH). Mass (EI), m/z (I*_relat_*.(%)): 331.3840 [M]^+^(74). C_17_H_15_F_2_N_3_S. ^1^H NMR (DMSO-d_6_, 300 MHz,): δ 1.36 (3H, t, *J =* 7.3, CH_3_CH_2_), 3.00 (3H, d, *J =* 4.4, NCH_3_), 3.15 (2H, q, *J =* 7.3, CH_3_CH_2_), 7.53 (1H, dt, *J =* 10.8, 8.5, FPhH6), 7.67 (1H, m, FPhH2), 7.70 (1H, dd, *J =* 2.0, 8.7, H6), 7.78 (1H, d, *J =* 2.0, H8), 7.92 (1H, ddd, J= 2.0, 8.0, 10, 12 FPhH5), 8.17 (1H, d, *J =* 8.7, H5), 8.37 (1H, br s, NH) ppm.

*2-(ethylthio)-N-methyl-7-[4-(trifluoromethyl)phenyl]quinazolin-4-amine*(11826087) Yeild 24%. Mp. 198-201ºС (EtOH). Mass (EI), m/z (I*_relat_*.(%)): 363.4011 [M]^+^(67). C_18_H_16_F_3_N_3_S. ^1^H NMR (DMSO-d_6_, 300 MHz,): δ 1.36 (3H, t, *J =* 7.3, CH_3_CH_2_), 3.01 (3H, d, *J =* 4.4, NCH_3_), 3.12 (2H, q, *J =* 7.3, CH_3_CH_2_), 7.75 (1H, dd, *J =* 1.9, 8.5, H6), 7.83 (1H, d, *J =* 1.9, H8), 7.84 (2H, d, *J =* 8.1, FPh), 8.04 (2H, d, *J =* 8.1, FPh), 8.22 (1H, d, *J =* 8.5, H5), 8.43 (1H, br s, NH) ppm.

*7-(2,4-difluorophenyl)-2-(ethylthio)-N-methylquinazolin-4-amine*(11826088)Yeild 47%. Mp. 185-8ºС (EtOH). Mass (EI), m/z (I*_relat_*.(%)): 331.3840 [M]^+^(62). C_17_H_15_F_2_N_3_S. ^1^H NMR (DMSO-d_6_, 300 MHz,): δ 1.35 (3H, t, *J =* 7.3, CH_3_CH_2_), 3.00 (3H, d, *J =* 4.4, NCH_3_), 3.13 (2H, q, *J =* 7.3, CH_3_CH_2_), 7.22 (1H, dt, *J =* 2.8, 8.4 , FPhH3), 7.39 (1H, dt, *J =* 2.8, 10.3, FPhH5), 7.52 (1H, d, *J =* 8.5, H6), 7.62 (1H, s, H8), 7.72 (1H, dt, *J =* 6.6, 8.9, FPhH6), 8.17 (1H, d, *J =* 8.5, H5), 8.41 (1H, br s, NH) ppm.

*8-chloro-2-(ethylthio)-7-fluoro-N-methylquinazolin-4-amine*(11826067)Yeild 71%. Mp. 153-6ºС (Hexane/EtOAc). Mass (EI), m/z (I*_relat_*.(%)): 271.7424 [M]^+^(34). C_11_H_11_ClFN_3_S. ^1^H NMR (DMSO-d_6_, 300 MHz,): δ 1.38 (3H, t, *J =* 7.3, CH_3_CH_2_), 2.99 (3H, d, *J =* 4.5, NCH_3_), 3.15 (2H, q, *J =* 7.3, CH_3_CH_2_), 7.43 (1H, t, *J =* 9.2, H6), 8.14 (1H, dd, *J =* 5.8, 9.2, H5), 8.57 (1H, br s, NH) ppm.

*2-(ethylthio)-6,8-difluoro-N-methylquinazolin-4-amine*(11826209) Yeild 56%. Mp. 173-6ºС (Hexane/EtOAc ). Mass (EI), m/z (I*_relat_*.(%)): 255.2881 [M]^+^(53). C_11_H_11_F_2_N_3_S. ^1^H NMR (DMSO-d_6_, 300 MHz,): δ 1.34 (3H, t, *J =* 7.3, CH_3_CH_2_), 2.98 (3H, d, *J =* 4.5, NCH_3_), 3.12 (2H, q, *J =* 7.3, CH_3_CH_2_), 7.63 (1H, t, *J =* 9.9, H7), 7.79 (1H, d, *J =* 9.7, H5), 8.39 (1H, br s, NH) ppm.
